# Profiling molecular regulators of recurrence in chemorefractory triple-negative breast cancers

**DOI:** 10.1186/s13058-019-1171-7

**Published:** 2019-08-05

**Authors:** Bradley A. Hancock, Yu-Hsiang Chen, Jeffrey P. Solzak, Mufti N. Ahmad, David C. Wedge, Dumitru Brinza, Charles Scafe, James Veitch, Rajesh Gottimukkala, Walt Short, Rutuja V. Atale, Mircea Ivan, Sunil S. Badve, Bryan P. Schneider, Xiongbin Lu, Kathy D. Miller, Milan Radovich

**Affiliations:** 1 Department of Surgery, Indiana University School of Medicine, 980 W. Walnut St. Room C312, Indianapolis, IN 46202 USA; 2 Department of Medical & Molecular Genetics, Indiana University School of Medicine, Indianapolis, IN 46202 USA; 3 Department of Microbiology and Immunology, Indiana University School of Medicine, Indianapolis, IN 46202 USA; 4 Department of Pathology and Laboratory Medicine, Indiana University School of Medicine, Indianapolis, IN 46202 USA; 5 Division of Hematology/Oncology, Department of Medicine, Indiana University School of Medicine, Indianapolis, IN 46202 USA; 6 Indiana University Melvin and Bren Simon Cancer Center, Indiana University School of Medicine, Indianapolis, IN 46202 USA; 7 Indiana University Center for Computational Biology and Bioinformatics, Indiana University School of Medicine, Indianapolis, IN 46202 USA; 8 Big Data Institute, Li Ka Shing Centre for Health Information and Discovery, Oxford, UK; 9 Department of Bioinformatics, ThermoFisher Scientific, Carlsbad, CA USA

**Keywords:** Triple-negative, Breast, Cancer, Recurrence, Chemoresistance, Relapse, TP53, SMAD2, MYC, TGF-beta

## Abstract

**Background:**

Approximately two thirds of patients with localized triple-negative breast cancer (TNBC) harbor residual disease (RD) after neoadjuvant chemotherapy (NAC) and have a high risk-of-recurrence. Targeted therapeutic development for TNBC is of primary significance as no targeted therapies are clinically indicated for this aggressive subset. In view of this, we conducted a comprehensive molecular analysis and correlated molecular features of chemorefractory RD tumors with recurrence for the purpose of guiding downstream therapeutic development.

**Methods:**

We assembled DNA and RNA sequencing data from RD tumors as well as pre-operative biopsies, lymphocytic infiltrate, and survival data as part of a molecular correlative to a phase II post-neoadjuvant clinical trial. Matched somatic mutation, gene expression, and lymphocytic infiltrate were assessed before and after chemotherapy to understand how tumors evolve during chemotherapy. Kaplan-Meier survival analyses were conducted categorizing cancers with TP53 mutations by the degree of loss as well as by the copy number of a locus of 18q corresponding to the SMAD2, SMAD4, and SMAD7 genes.

**Results:**

Analysis of matched somatic genomes pre-/post-NAC revealed chaotic acquisition of copy gains and losses including amplification of prominent oncogenes. In contrast, significant gains in deleterious point mutations and insertion/deletions were not observed. No trends between clonal evolution and recurrence were identified. Gene expression data from paired biopsies revealed enrichment of actionable regulators of stem cell-like behavior and depletion of immune signaling, which was corroborated by total lymphocytic infiltrate, but was not associated with recurrence. Novel characterization of TP53 mutation revealed prognostically relevant subgroups, which were linked to MYC-driven transcriptional amplification. Finally, somatic gains in 18q were associated with poor prognosis, likely driven by putative upregulation of TGFß signaling through the signal transducer SMAD2.

**Conclusions:**

We conclude TNBCs are dynamic during chemotherapy, demonstrating complex plasticity in subclonal diversity, stem-like qualities, and immune depletion, but somatic alterations of TP53/MYC and TGFß signaling in RD samples are prominent drivers of recurrence, representing high-yield targets for additional interrogation.

**Electronic supplementary material:**

The online version of this article (10.1186/s13058-019-1171-7) contains supplementary material, which is available to authorized users.

## Background

Triple-negative breast cancers (TNBCs) are defined by lack of overexpression of the estrogen receptor (ER), progesterone receptor (PR), and human epidermal growth factor receptor-2 (HER-2). Although it comprises only 15–20% of all breast cancer cases, TNBC is responsible for a disproportionately high rate of morbidity and mortality compared to other breast cancer subtypes [1]. Neoadjuvant chemotherapy (NAC) is indicated for the treatment of stage I–III TNBC. Approximately 25–30% of patients achieve a pathologic complete response (pCR) after NAC and experience an excellent outcome: a 3-year overall survival of 94%. In contradistinction, roughly 50% of patients who have residual disease (RD) in the breast or lymph nodes will present with recurrent disease within 3–4 years and have a corresponding 3-year overall survival of only 68% [2, 3]. Despite significant advances in the treatment of breast cancer, no targeted therapies exist for these individuals.

Recurrence is the key factor driving mortality in patients who present with early-stage TNBC. Discerning aggressive cancers with a high risk-of-recurrence and developing tailored therapies for their treatment signifies a critical frontier in the clinical management of TNBC. In addition, recurrent cancers are highly refractory to chemotherapy, indicating a significant unmet need for the discovery of efficacious targeted agents. Mapping evolution during chemotherapy and recurrent molecular features of RD samples and relating them to relapse represents a high-yield approach for determining critical drug targets. To these ends, we conducted a comprehensive genomic analysis to unearth important molecular processes critical to the biology of RD. From a phase II post-neoadjuvant clinical trial of patients with TNBC, we procured pre-NAC biopsies, matched RD specimens, and survival data. Herein, we describe the complex molecular evolution during chemotherapy and identify novel molecular entities driving relapse of high-risk TNBC.

## Methods

### Samples

**Fig. 1:**
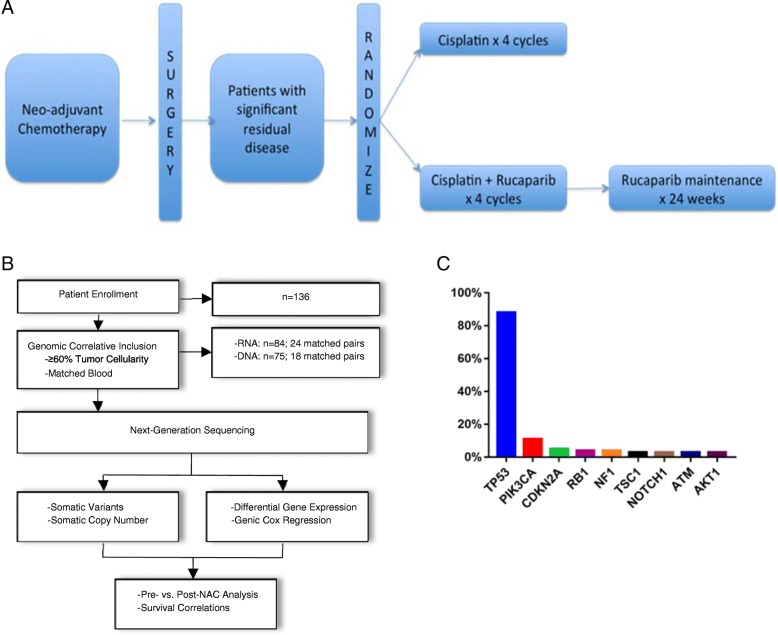
Genomic analysis of BRE09-146. **a** Clinical trial schema. Patient samples were collected before and after neoadjuvant chemotherapy. **b** CONSORT diagram of genomic analysis. **c** Recurrently mutated genes in BRE09-146

We derived tumor samples from the phase II clinical trial BRE09-146: PARP Inhibition After Preoperative Chemotherapy in Patients With Triple-Negative or ER/PR+, HER2 Negative Breast Cancer With Known BRCA1/2 Mutation (clinicaltrials.gov; NCT01074970). As part of the pre-planned correlative studies for this trial, tumor samples were collected at the time of surgery along with diagnostic biopsies at the time of diagnosis prior to NAC (Fig. 1a, b). All tumor samples were derived from archived tissue in formalin-fixed paraffin-embedded (FFPE) blocks. H&E slides were made from each block and reviewed by a pathologist (S.B.) to determine tumor cellularity and total lymphocytes. Evaluation of total lymphocytes was selected prospectively and retained for the analysis because lymphocytes not affiliated with the tumor or stroma and intratumoral tumor-infiltrating lymphocytes (iTILs) are relatively rare. Therefore, total lymphocytes were deemed a reasonable surrogate for stromal TILs, which have prognostic value in treatment-naïve, early-stage TNBCs [4]. Only those tumor tissues with ≥ 60% tumor cellularity were used for molecular studies. Of note, 60% tumor cellularity is the defined cutoff used by The Cancer Genome Atlas (TCGA). DNA and RNA were extracted from three 10-μm sections using the Qiagen All Prep DNA/RNA FFPE Kit along with Qiagen deparaffinization solution. RNA was quantified using the Qubit RNA Assay Kit (Life Technologies), and RNA sized using the Agilent TapeStation 2200 along with the R6K Screen Tape Kit. All samples and studies were approved by the Indiana University Institutional Review Board (IRB).

### Next-generation RNA sequencing

To enrich for the non-ribosomal RNA transcriptome, RNA samples were first depleted of ribosomal RNA using the Low Input Ribominus Kit (Life Technologies). External RNA Controls Consortium (ERCC) spike-in controls (Pool 1) were introduced for all samples (Life Technologies). RNA libraries were constructed using the Ion Total RNA-Seq Kit for AB Library Builder System (Life Technologies) per manufacturer’s instructions, except for a single modification where enzymatic shearing of the RNA was performed only for 15 s due to the already fragmented nature of RNA derived from FFPE. Libraries were barcoded using the IonXpress RNA-Seq Barcode 1-16 Kit (Life Technologies), and libraries were quantified using the Agilent TapeStation 2200 along with the DNA D1K Kit. Libraries were diluted to a concentration of 11 pM prior to templating and emulsion PCR using the Ion Template OT2 200 v2 kit along with Ion OneTouch 2 instrument (Life Technologies). Templates were quantified using the IonSphere Quality Control Kit (Life Technologies). Samples were sequenced on an Ion Proton Next-Generation Sequencer using the Ion Proton PI chip and the Ion PI Sequencing 200 v2 kit (Life Technologies). Samples were sequenced using two RNA-Seq libraries/samples per chip to an average of 30–40 million reads per sample. RNA sequencing data is to be submitted to Gene Expression Omnibus.

### Targeted DNA sequencing

Highly cellular DNA and matched samples were sequenced across the 134 genes included in the Oncomine Cancer Panel (Life Technologies). Library preparation was conducted using 10–20 ng of starting input. Emulsification PCR on amplicon libraries and sequencing chip loading were conducted on using Ion Chef (Life Technologies). Semi-conductor sequencing was performed using the Ion Proton and accompanying PIv3 chip (Life Technologies). Libraries were sequenced in high coverage, >1000×. DNA sequencing data is to be submitted to Gene Expression Omnibus.

### RNA-Seq bioinformatics

Raw reads from the Ion Proton sequencer were converted from unaligned BAM format to fastq using the bam2fastq utility. Reads were mapped to hg19 using the Star RNA-Seq Aligner. The NCBI Refseq database was used as the gene model. Read counts were normalized using RPKM. Default input settings were utilized for pathway analysis in Ingenuity Pathway Analysis. Only genes, RNAs (excluding miRNAs), and proteins were included in the Upstream Regulator output.

### Variant and copy number calling

Variant calling was performed using Torrent Suite version 5.0.3.2. All variants identified were present in tumor and absent in the matched normal genome. In addition, somatic variants were manually reviewed using Integrated Genomics Viewer (IGV) for systematic error. A reference range for normal heterozygosity of TP53 was determined using three common single nucleotide polymorphisms (SNPs) in TP53. The reference range was established using the mean of allele frequencies for heterozygous calls ± 3SD. Three standard deviations were utilized to account for DNA degradation caused by formalin processing. Copy number calls for tumor and normal genomes were derived from 70 genes in the Oncomine Cancer Panel using Ion Reporter (Life Technologies), which utilizes the Hidden Markov Method. Gains and losses were called based upon the corresponding per-assay reference range, which was established using the normal genomes. Cutoffs were defined as the mean copy number from the normal genomes ± 3 SD. Copy number greater than 4.99 was considered amplified, representing a conservative threshold. Genomic and transcriptomic data submission to Gene Expression Omnibus is pending.

### Statistics

Analysis of variance (ANOVA) was used for differential gene expression and copy number analyses. Upstream regulator analysis calculates the overlap of regulator networks using Fisher’s exact test (www.qiagenbioinformatics.com). Differential lymphocytic infiltrate and single gene expression analyses were analyzed using Student’s *t* test. Univariate and multivariate log-rank analysis was used to determine survival advantages. Age, lymph node status, and RCB category were selected as covariates. Age was not deterministic upon univariate analysis. RCB category (I/II vs. III) and lymph node positivity (positive vs. negative) were analyzed as categorical variables (Additional file 10: Table S9).

## Results

### Genomic analysis of chemoresistant TNBCs in BRE09-146

We compiled genomic and transcriptomic data across a cohort of patients with TNBC in a phase II clinical trial to obtain a comprehensive view of the molecular factors that are associated with chemoresistance and patient outcomes. BRE09-146 (NCT01074970) was a post-neoadjuvant clinical trial for patients with chemoresistant triple-negative or BRCA1/2-mutated hormone receptor (HR)-positive breast cancer (Fig. 1a). This trial evaluated the poly-a-ribose polymerase (PARP) inhibitor, rucaparib (Clovis), in combination with cisplatin versus cisplatin alone in the post-neoadjuvant setting and utilized 2-year disease-free survival (DFS) as the primary endpoint. Prior to enrollment, all 135 patients received an anthracycline, taxane, or both agents concurrently, followed by surgery and had RD per pre-defined protocol criteria. No prior cisplatin was allowed. We defined RD as having at least one of the following: a Miller-Payne response in the breast of 0–2, a residual cancer burden (RCB) classification of II or III, residual carcinoma in one or more regional lymph nodes that would meet AJCC 6th edition criteria for N1–N3 disease, or residual invasive disease in the breast measuring at least 2 cm. The results of the primary endpoint of the trial were negative, and hereditary BRCA1/2 mutation status did not improve DFS [5]. In addition, pharmacokinetic analyses revealed suboptimal dosing of rucaparib as patients received roughly 10% of the FDA-approved 600 mg twice daily [5]. Thus, the trial was considered single-armed for the genomic analysis. Patients excluded from all analyses included 6 of 135 individuals who were HR+. We collected FFPE-derived tumor tissue at the time of diagnosis (pre-NAC) and definitive surgery (post-NAC) along with blood for somatic mutation calling. Whole-transcriptome sequencing was performed on samples with high tumor cellularity (≥ 60%), corresponding to 84 patients on trial. This included 24 matched cases that were profiled before and after NAC. In addition, targeted next-generation sequencing was performed across the exons of 134 cancer-related genes using the Ion Oncomine Cancer Panel (Additional file 2: Table S1). Tumors with adequate cellularity tended to have higher RCB scores as compared to those with lower cellularity, indicating a selection bias towards more aggressive cancers (Additional file 3: Table S2). We collected (> 1000X) somatic mutation and copy number data, normal blood, and clinical follow-up data for 75 patients with highly cellular tumor samples (≥ 60% tumor cellularity), including 18 matched pairs (Fig. 1b). A high-level review of our genomic data revealed many previously known somatic mutations common to TNBC.TP53 and PIK3CA were the most recurrently mutated genes at frequencies of 88% and 11%, respectively, with a number of additional genes mutated infrequently (Fig. 1c). In addition, we generated copy number data across 70 genes with loci on 21 chromosomes and averaging 3 gene targets per chromosome (no targets on chr6 and chr21). We constructed reference ranges for diploidy using data from the normal leukocyte-derived genomes on a per-assay basis and observed marked copy number aberration, including nearly universal gains on 1q, 8q, and 10p with frequent amplifications in MCL1 (21%), MYC (24%), and GATA3 (11%) (Additional file 1: Figure S1). The most recurrently lost gene was BRCA2 (68%, 13q13), lost commonly in conjunction with FLT3 (13q12) and RB1 (13q14). These data are largely in agreement with other large-scale sequencing studies conducted in TNBC, thus verifying the validity of our molecular data [6–13] (Additional file 4: Table S3).

### Genomic evolution in chemoresistant TNBC

**Fig. 2:**
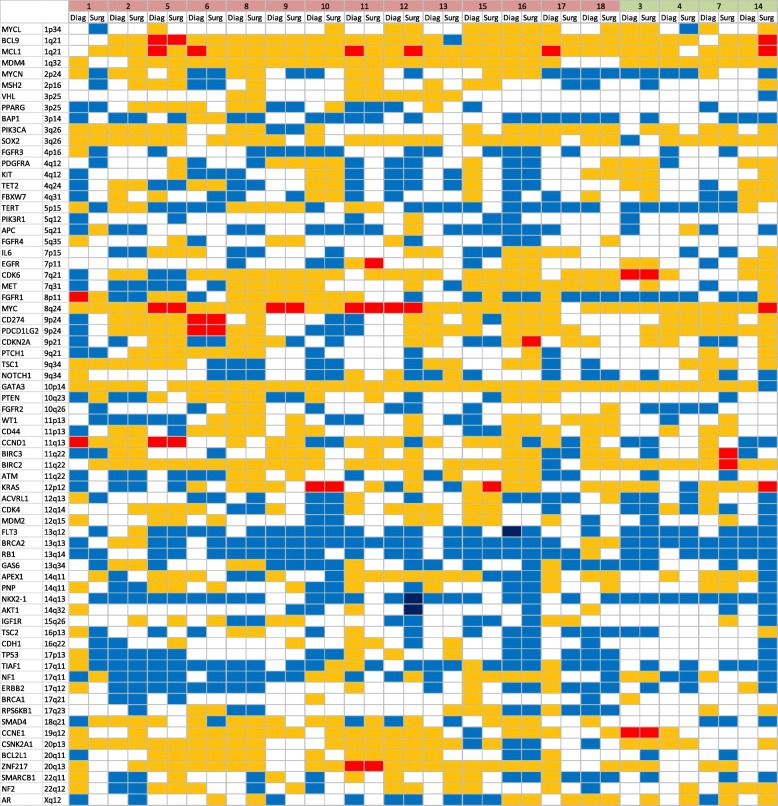
Pairwise analysis. Copy number heatmap across 18 pairs. “Diag” denotes diagnosis/pre-chemotherapy; “Surg” denotes definitive surgery/post-chemotherapy. Gains/losses are denoted as follows: high-level amplification (red, 5.0+ copies), gain (yellow), normal (white), minor loss (royal blue), extensive loss (dark blue, < 0.5). Pairs in red recurred. Pairs in green remained recurrence free

Multiple compelling lines of evidence exist as to how some tumors are able to escape the effects of chemotherapy while others are highly sensitive. Clonal evolution, epithelial-to-mesenchymal transition (EMT), and tumor-initiating capacity/stemness are tightly correlated with chemoresistance [14]. Examining patterns of how cancer genomes change during the course of chemotherapy and then correlating the respective changes to recurrence can reveal deterministic factors of recurrence that are acquired under selective pressure. To examine the evidence of this phenomenon in our validated dataset, we leveraged our matched pairs and hypothesized that (1) clonal evolution would be identified by the robust acquisition of new mutations and copy number variants after chemotherapy and (2) recurrent patterns would be correlated to relapse. Gene-level mutation profiles across the 18 available cases were unrevealing and mirrored frequently mutated genes in the larger cohort with common mutations in TP53 and PIK3CA. In the matched analysis, 13 of the 18 cases had identical mutation profiles before and after chemotherapy. In the other five cases, we observed the acquisition of only one FGFR3 mutation and individual losses of point mutants in CDKN2A, TP53, ATM, BRCA2, and PNP. A paired copy number analysis revealed the chaotic acquisition of gains and losses in post-NAC tissues, yet we did not identify commonly recurrent loci (Fig. 2). Previously known copy number alterations characteristic to TNBC, such as gains in 1q and 8q, were already present at diagnosis and did not evolve through the course of chemotherapy. Twenty-eight occurrences of high-level amplification (≥ 5 copies) were detected in expected genes such as MCL1 (*n* = 6) and MYC (*n* = 5). Interestingly, the evolution of gene amplification during chemotherapy was observed in KRAS (*n* = 2), MCL1 (*n* = 2), BCL9 (*n* = 1), EGFR (*n* = 1), MYC (*n* = 1), CDKN2A (*n* = 1), BIRC2 (*n* = 1), and BIRC3 (*n* = 1). In addition, we unexpectedly observed loss of gene amplification in the post-NAC time point in 6 cases, indicating clonal extinction (Fig. 2). We refined our hypothesis to analyze if cancers that evolved amplification events in robust oncogenes would be prone to relapse. In individuals, 14 of the 18 cancers profiled recurred, and 4 did not. While the statistical power in this analysis is limited, we surprisingly identified 2 of the 4 cancers that had durable responses to NAC (non-relapsers) evolved high-level amplifications in known oncogenes. Moreover, in one example, a MYC and KRAS amplification co-evolved together, but this phenomenon did not empower recurrence. These data together suggest chemoresistant TNBC genomes are highly unstable during chemotherapy, generating dramatic copy number variation (CNV) resulting in the rapid development of dominant subclones. However, despite the evolution of provocative aberrations such as high-level MYC amplification, detecting loci significant to recurrence was elusive.

### Immune depletion and stem-like enrichment after chemotherapy

**Fig. 3:**
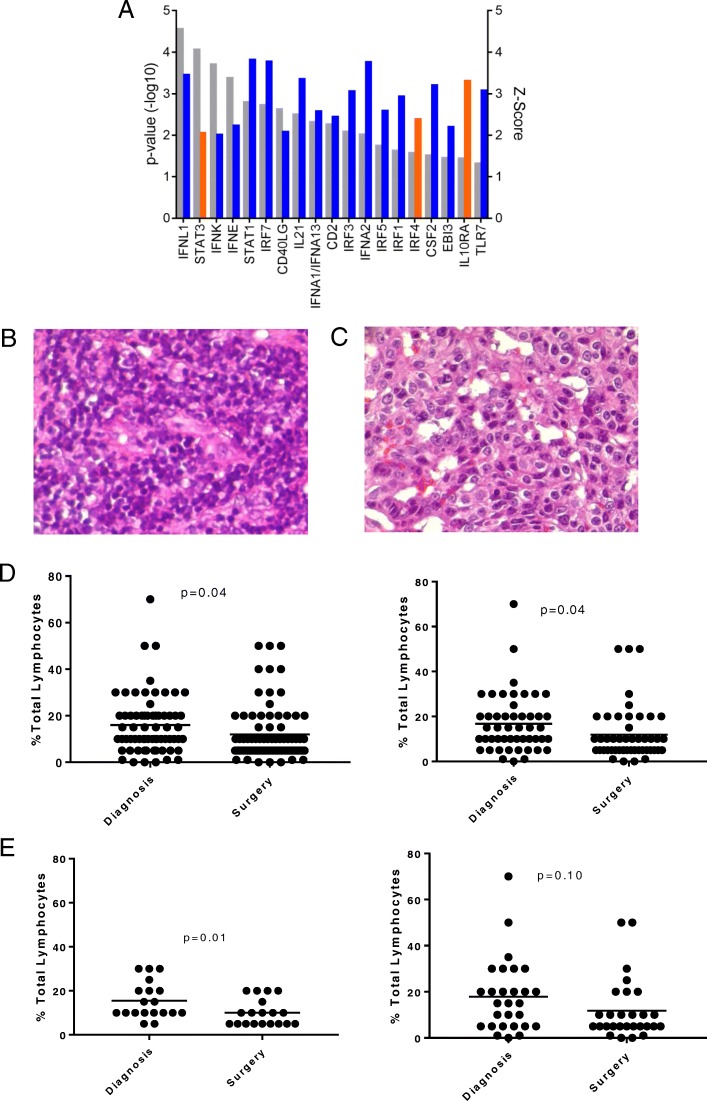
Immune depletion analysis. **a** Immune upstream regulator analysis. Differential in silico regulator activity as called by Ingenuity Pathway Analysis. Regulator confidence (*p*-values) are relayed in gray with accompanying *Z*-scores relayed in orange (upregulated) or blue (downregulated) (*n* = 24 pairs). **b**, **c** Hematoxylin and eosin-stained tissues from before (**b**) and after (**c**) chemotherapy demonstrating a decrease in total lymphocytes. **d** Unpaired (left) and paired (right) total lymphocyte% as called before and after surgery (unpaired: *n* = 67,83, *p* = 0.04; paired: *n* = 49, *p* = 0.04). **e** Paired total lymphocyte% in non-relapsers (left) and relapsers (right) (non-relapse: *n* = 20, *p* = 0.01; relapse: *n* = 28, *p* = 0.10). **d** and **e** were performed with Student’s *t* test

TNBC genomes are chaotic and complex, making the discovery of genotype/phenotype correlations arduous. Cogent to our analysis, genomic drift was readily apparent, yet cumulative and convincing proof of how it leads to recurrence was not. We hypothesized that by comparing gene expression profiles before and after NAC in BRE09-146, we could identify critical factors driving resistance. Using an ANOVA analysis, we detected 878 genes with a differential expression ≤ 10% FDR (Additional file 5: Table S4). We condensed our gene set by performing an upstream regulator analysis in Ingenuity Pathway Analysis (IPA), a prediction tool which analyzes differential gene expression data against a comprehensive database of published interactions to predict up- or downregulation of entities upstream of multiple gene expression differences (Ingenuity Systems, www.ingenuity.com). We identified 37 regulators with potent changes between time points (*p* ≤ 0.05, *Z*-score > 2, Additional file 6: Table S5). Strikingly, 19 of the 37 (51%) regulators indicated a sharp drop-off in immune activity with a particular enrichment in interferon signaling. Deactivation of the type-III interferon, interferon lambda-1 (IFNL1), was associated with the highest confidence of all regulators (Fig. 3a). Recently discovered, the type-III interferon family is associated with immune-mediated anti-tumor effects and shows a favorable toxicity profile in pre-clinical models [15]. These results are in accordance with a similar analysis of the I-SPY 1 trial in which decreased immune activity was identified in a paired analysis of tumor tissue assessed before and after chemotherapy, though it should be noted that this dataset contained HR+ and HER2+ tumors with few TNBCs, leaving an important need, for which our study fulfills [16]. To further elucidate our observation of immune depletion, we performed immune deconvolution using CIBERSORT across 49 and 57 samples taken before and after chemotherapy, respectively [17]. CIBERSORT is an analytical tool that uses gene expression data to provide an estimation of the abundances of member cell types in a mixed cell population [17]. Using the input of 9407 and 8033 genes, only 3 and 5 samples passed quality control in CIBERSORT. This was due to the extreme paucity of immune genes expressed in our dataset; 21% and 17% of the 547 genes used in CIBERSORT’s LM22 immune panel were detected, prohibiting a canonical CIBERSORT analysis. These QC assessments using CIBERSORT are in agreement with cutoffs from other breast cancer studies [18]. As a separate ad hoc study, 86 of 547 immune-related genes interrogated in a standard CIBERSORT analysis were detected between the two time points, and 22 were differentially expressed (*p* < 0.05). Not surprisingly, all but one of the overlapping significant genes were downregulated. Moreover, Granzyme A (GZMA) and Perforin 1 (PRF1), which have been validated as surrogates of immune activity in cancer, were not expressed in either dataset [19].

In addition to immune signals, we observed evidence indicating the development of a stem cell-like phenotype post-chemotherapy in the paired upstream regulator analysis. Significant predicted changes in regulator activity of MAPK1, MAP 4K4, STAT3, STAT1, and CD24 were detected during the course of chemotherapy with increases in activity for the first three and decreases in the remnant. MEK-ERK and JAK-STAT are both central and actionable processes related to breast cancer stem cell signaling in TNBC [20–26]. Of particular interest was STAT3/STAT1. These regulators work in inverse directions to establish a stem cell phenotype and repress the immune system simultaneously, suggesting this axis may be a potent resistance mechanism [27, 28].

### Suppression of the immune system and recurrence

Tumor-infiltrating lymphocytes have been shown to predict pCR and long-term outcome in TNBC [29–31]. Based on the predicted deactivation of immune regulators after chemotherapy from our gene expression analysis, we hypothesized that we would observe a corresponding downregulation of total lymphocytic infiltrate in a pre- and post-chemotherapy analysis of our dataset. We also hypothesized that pre- and post profiles of cancers that recurred would be distinct from those that did not. The analysis of 67 and 83 pre- and post-chemotherapy tumors revealed a parallel decrease in total lymphocytes in accordance with our sequencing data (Fig. 3d, *p* = 0.04). This finding was also significant when the cohort was reduced to only matched pairs (*n* = 49, *p* = 0.04; Fig. 3d). To understand how this phenomenon relates to recurrence, we analyzed cancers that recurred independently from those that did not. We observed a significant decrease in lymphocytic infiltrate in the cancers that did not recur in contrast to those that were associated with recurrence, although we note the data is trending similarly in both sets, most probably aligning our data with that of others suggesting decreases in infiltrate are not significant with respect to recurrence (Fig. 3e) [32]. Additionally, we extended our investigation to include outcome data. Categorical and continuous analyses associating lymphocytic infiltrate from samples obtained both at diagnosis and surgery were negative.

### Graduated inactivation of p53 and relapse

**Fig. 4:**
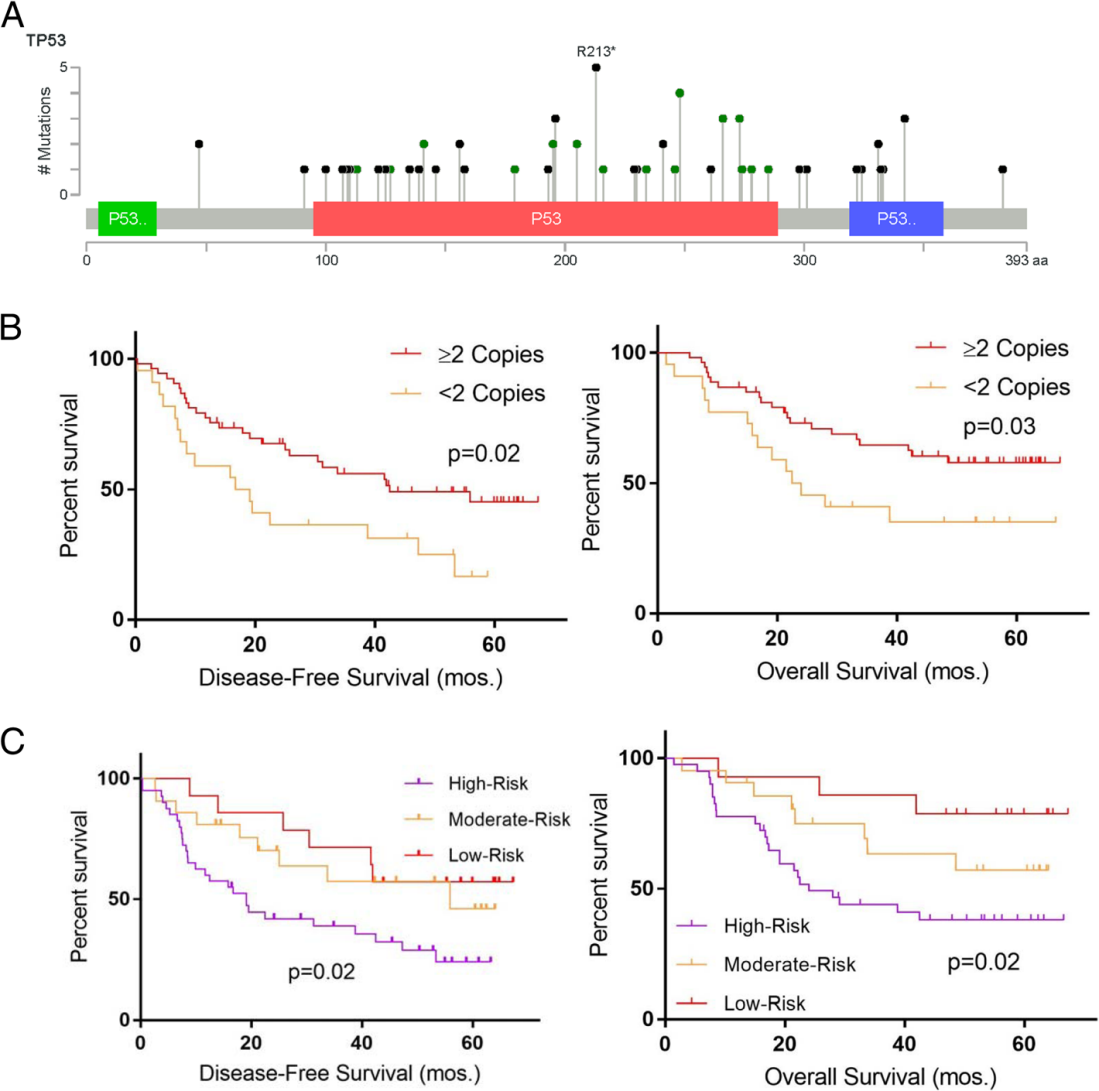
Graduated p53 inactivation and outcome in BRE09-146. **a** Lollipop plot of TP53 mutations in BRE09-146. **b** Kaplan-Meier disease-free and overall survival analysis of TNBCs with < 2 copies vs. ≥ 2 copies of TP53 (*n* = 75, 53 vs. 22; DFS: HR = 2.07 (1.03–4.17), *p* = 0.02; OS: HR = 2.07 (0.96–4.48); *p* = 0.03). **c** Three-way Kaplan-Meier analysis of disease-free and overall survival analysis comparing outcomes based upon risk stratification predicted by the degree of genomic loss of TP53 (14 vs. 21 vs. 40; DFS: *p* = 0.02; OS: *p* = 0.02)

The vast majority of TNBCs harbor TP53 mutations. Despite well-characterized and robust involvement in TNBC, somatic TP53 status has virtually no clinical utility. We were struck by the diversity in p53 mutations present our dataset and the nature of these mutations on a per-sample basis. Some tumors harbored extensive involvement, displaying such features as LOH or copy loss of p53, while others contained only subclonal point mutations [10]. These aspects of p53 inactivation are rarely acknowledged. Thus, we hypothesized that variability in the depth of inactivation may have a significant impact upon the course of an individual cancer. To take an inclusive survey of genomic correlations between p53 inactivation and survival in BRE09-146, we integrated follow-up data along with p53 somatic mutation and copy number data. We also combined pre-NAC and post-NAC samples into one conglomerate containing 75 patients on the basis that we did not observe significant deviation in TP53 mutations, allele frequencies, or copy loss between time points. Importantly, we corrected all mutated allele frequencies and copy numbers for tumor percentage in the tissue. After merging the data, the majority (91%) of patients contained at least one type of aberration in TP53. The mutation class distribution (i.e., missense, nonsense, etc.) revealed the heterogeneity expected in TNBC and other solid tumors (Fig. 4a). We first tested our hypothesis based solely on copy losses in TP53. Twenty-two (29%) of cases presented with abnormally low copy number. This frequency represents a moderate difference from the treatment-naïve TCGA (67%). Our implementation of a conservative confidence interval to adjust for formalin fixation may partially account for this particular difference. Despite this phenomenon, we did not observe this effect across other targets in the copy number analysis. Intriguingly, when we compared the outcomes for cancers with copy loss of TP53 versus those without loss of TP53, we observed significantly decreased DFS and OS for those with tumors that had lost TP53 (Fig. 4b, Additional file 10: Table S9). We concluded that loss of this locus of chromosome 17p is an important event governing the aggressiveness of TNBC. An important covariate of this analysis was that only 2/22 tumors with TP53 copy loss did not harbor a concomitant TP53 point mutation. Based on these findings, we hypothesized that the increased tumor aggression we observed was driven by the synergizing effects of a point mutation plus additional copy loss. We broadened our hypothesis, postulating that other factors such as loss-of-heterozygosity, aneuloploidy, and subclonality may be congruently relevant to outcomes, resulting in the possibility of several different degrees of functional loss. We opted to isolate from this milieu two categories representing extreme ends of the continuum: (1) those with no TP53 mutation or only a subclonal mutation, which we hypothesized to have low risk-of-relapse, and (2) inversely, those that had a mutation and some other compounding event such as loss-of-heterozygosity or 17p copy loss, which we called “high-risk.” We additionally hypothesized the risk-of-relapse for all other cancers would be “moderate.” Of note, we set the normal reference range for allele frequency at 38–59% based upon a confidence interval obtained from normal samples. However, 25% was determined to be a superior cutoff for subclonal mutations for the purpose of avoiding ambiguity caused by tumors with high copy number (maximum = 4). A Kaplan-Meier survival analysis comparing the DFS and OS of these three subgroups revealed a step-wise graduated survival outcome in accordance with the predicted degree of p53 activity (Fig. 4c, Additional file 10: Table S9). Similar observations have been reported previously clinically and pre-clinically [33, 34].

### Compound TP53 mutation status and MYC signaling

**Fig. 5:**
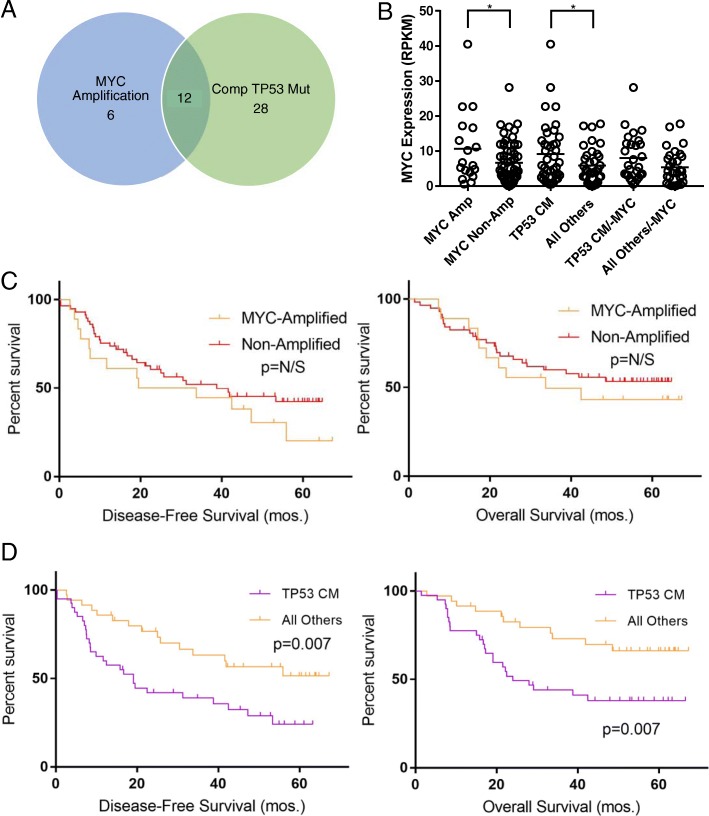
Compound TP53/MYC status and survival. **a** Venn diagram displaying overlap of a total of 46 TNBCs with MYC amplification and/or compound mutation of TP53. **b** MYC RNA expression in TNBCs (1) with MYC amplification vs. no amplification , (2) TP53 compound mutations (TP53 CM) vs. all others (*p < 0.05; *n* = 75, Student’s two-tailed *t* test), and (3) TP53 compound mutations vs. all others with all samples with MYC amplification removed. **c** Kaplan-Meier disease-free and overall survival analysis comparing outcomes of TNBCs with MYC amplification vs. no amplification (*n* = 75, 18 vs. 57). **d** Kaplan-Meier survival analysis of disease-free and overall survival comparing TP53 compound mutation (*n* = 75, 40 vs. 35; DFS: HR = 2.30 (1.26–4.20); *p* = 0.007; OS: HR = 2.55 (1.31–5.00); *p* = 0.007)

To better understand the molecular processes driving tumors with extensive p53 inactivation and inferior outcome, we conducted a differential gene expression analysis between cancers with compound loss and all others. We were surprised by a sheer global upregulation of gene expression for cancers with compound p53 mutations as compared to the remainder. Of the roughly 2000 genes differentially expressed with a confidence of *p* = 0.05, only 1% were downregulated. When we applied a FDR of 10%, 508 genes remained, and 100% were overexpressed in the compound group (Additional file 7: Table S6). This type of transcriptional amplification has been previously reported as a direct footprint of MYC activity [35, 36]. An upstream regulator analysis conducted using the FDR-corrected dataset corroborated this finding, predicting MYC signaling as significantly upregulated (*p* = 1.48 × 10^−5^, *Z*-score = 4.177, Additional file 8: Table S7). Pan-cancer analyses have revealed that cancers with MYC amplifications commonly have co-occurring mutations in TP53. Thus, we investigated the covariate overlap of these drivers in our dataset [37]. As displayed by the Venn diagram in Fig. 5a, a moderate number of cancers (12/46 (26%)) in our dataset shared these two mutations. Additionally, we interrogated associations between MYC expression and MYC amplification, as well as TP53 status. We hypothesized MYC amplification would be associated with increased MYC expression and that TP53 mutation would not, exerting its effect in a downstream reciprocal fashion as has been observed elsewhere [38]. In our analysis, we observed statistically significant correlations between MYC expression and MYC amplification status as well as TP53 mutation status (Fig. 5b). However, when cancers with MYC amplification were removed, the latter correlation disbanded, confirming our hypothesis. To further differentiate this from MYC amplification, we conducted a survival analysis comparing MYC-amplified TNBC to non-amplified TNBC, for which there was no survival correlation (Fig. 5c). In contrast, a survival analysis comparing cancers with compound TP53 mutation versus all others revealed a significant recurrence and survival disadvantage (Fig. 5d, Additional file 10: Table S9). These results cumulatively suggest a complex interplay in TNBC, as has been observed in other MYC-driven cancers, by which deep losses in TP53 enable oncogenic MYC signaling, which in turn drives recurrence and mortality [38].

### Exploratory analysis of recurrence and copy number variation in RD tumors

**Fig. 6:**
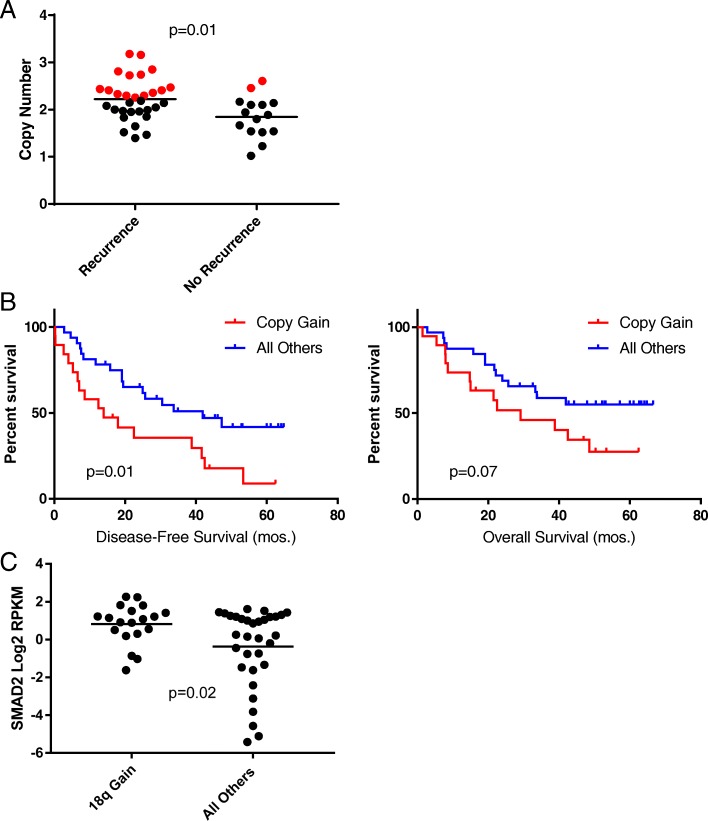
Gains in 18q determine recurrence and overexpression of SMAD2. **a** SMAD4 copy number in recurrent vs. non-recurrent cancers by ANOVA. Red and black dots represent gains and normal/loss, respectively (*n* = 46; Student’s *t* test). **b** Kaplan-Meier disease-free and overall survival of SMAD4 gains versus all others (*n* = 51, 19 vs. 32; DFS: HR = 2.30 (1.08–4.87); *p* = 0.01; OS: non-significant). **c** SMAD2 overexpression association with 18q gains by ANOVA (*n* = 51)

As evidenced by our results and work of others, copy number profiles in TNBC genomes are highly dysregulated. With the exception of relatively rare focal amplifications, which are reasonably recurrent largely in MYC and MCL1, copy number gains and losses are frequently unexplored as drivers of recurrence in TNBC. We hypothesized that differential CNV in cancers that recurred may impart a metastatic or recurrence-prone phenotype. We opted to use a two-part statistical model to best utilize power and minimize risk of artifact. First, ANOVA was used to detect differences in absolute copy number present between cancers that recurred and those that did not. Loci with differential absolute copy number were grouped according to gain/loss status and interrogated for time-to-recurrence using a Kaplan-Meier survival analysis. We considered recurrent loci positive if they passed both steps. The results of the ANOVA analysis indicated 5/70 assessed loci were statistically different between recurrent and non-recurrent tumors (*p* < 0.05; not shown). However, only gains in a locus containing the SMAD4 (mothers against DPP homolog4) gene on the long arm of chromosome 18 (18q) also predicted DFS (Fig. 6a, b, Additional file 10: Table S9). SMAD4 is a signal transducer of the TGFß signaling pathway, which has a dual role as a tumor suppressor in early tumorigenesis, but a contrasting pro-metastatic role late in cancer development [39]. Moreover, TGFß signaling has been correlated with chemoresistance in TNBC [40]. Gains in SMAD4 occurred at a reasonably similar frequency in the treatment-naive TCGA (25%), validating our sequencing results.

We also hypothesized that a parallel upregulation of SMAD4 RNA expression would be observed in concert with gains in SMAD4 to drive the recurrent phenotype. However, upregulation of SMAD4 was not seen. We reasoned alternatively SMAD4 may be gained in concert with other genes on 18q that portend DFS. We filtered our RNA expression dataset for genes located on 18q21.1 and 18q21.2, which corresponds to a region of approximately 10 Mb centered around SMAD4. From our surgical samples, we identified 19 expressed genes in the region (Additional file 9: Table S8). Interestingly, 3 expressed SMADs exist in the aforementioned region: SMAD2, SMAD4, and SMAD7. All 3 genes were gained in high concordance (95%) with SMAD4 in the 135 tumors from the TCGA, indicating these genes are gained and lost concomitantly [7]. Differential gene expression of genes in the region revealed that SMAD2 was the only overexpressed protein-coding gene, suggesting gains in this region of 18q may cause dysregulation of TGFß signaling through upregulation of SMAD2 (Fig. 6c). Interestingly, SMAD2 upregulation has been implicated in the recurrence of TNBCs previously [41].

## Discussion

Chemorefractory TNBCs are associated with a dichotomous disease course. Half of the patients with RD will be disease-free longterm. In stark contrast, recurrence and death rapidly overtake the remainder, in large part due to the lack of therapies that are effective against this aggressive disease. To guide drug discovery, we interrogated trends in the molecular underpinnings of TNBC RD tumors and revealed several key findings with respect to recurrence.

We first hypothesized that patterns in how TNBCs evolve during treatment would expose critical mechanisms of resistant cancers. Intriguingly, we observed little evidence of genomic instability as measured by the acquisition of point mutations and indels, while dynamic copy number changes, including high-level focal amplifications, were acquired during the course of chemotherapy. However, we detected no correlation between clonal evolution and recurrence. Our observations are consistent with data from others, demonstrating that the bulk of the genomic instability of TNBC genomes is largely driven by somatic copy number changes that are enabled by loss of TP53 and seem to emerge in rapid bursts [8, 12, 42]. In addition, our study bolsters other findings, which have demonstrated clonal shifting during chemotherapy, yet failed to identify mutations in specific loci that portend recurrence and instead suggest the recurrent phenotype in TNBC is possibly driven by cell plasticity [43, 44]. We note the limitations of a targeted panel to detect broad clonal evolution and support analyses such as a copy number-driven mutated pathway analysis.

Complementary RNA sequencing of matched pairs revealed important phenotypic context, including depletion of the immune microenvironment and upregulation of markers related to stemness, which validates what others have observed previously in the I-SPY1 trial evaluating all breast cancer subtypes [16]. STAT1/3 signaling, MAPK signaling, and a decrease in CD24 activity together likely represent cellular reprogramming or epithelial debulking, resulting in the enrichment of a stem cell-like phenotype, which corroborates what others have seen pre-clinically and would be expected to drive chemoresistance and metastasis [20–23, 26, 27]. Likewise, previous publications indicate immune infiltration portends pCR and outcome in TNBC [29–31]. Our paired interrogation of lymphocytic infiltrate before and after chemotherapy revealed significant depletion of infiltrate after chemotherapy, validating our transcriptomic data. We detected significant depletion in tumors that did not recur in contrast to those that did, for which no correlation was seen. While intriguing, given that the lymphocyte percentage was lower in both groups post-NAC and the borderline results observed for the recurring group, we suspect that lymphocytic depletion is characteristic of all TNBC RD tumors, in concordance with other findings [32]. In contrast, we did not detect trends between immune infiltration in RD tumors and recurrence [32, 45]. This may be explained in a few ways. First, the size of our dataset may be problematic given the low frequency (10%) of lymphocyte-predominant residual tumors [45]. Second, the administration of post-neoadjuvant cisplatin may change the association between immune infiltrate and recurrence.

Strictly from our RD samples, we revealed trends with recurrence in a confluence of regulators: TP53, MYC, and TGFß signaling. We first established a graduated role for p53 in the governance of relapse and mortality in chemorefractory TNBC. Patients in our study were stratified into three prognostically relevant subgroups, based on the predicted level of p53 activity in their tumor. Inactivation of p53 is known to be one of the most predominant mechanisms of post-target resistance to chemotherapy [46]. TP53 mutation has also been reported as a predictive marker for resistance to adjuvant chemotherapy in TNBC, but this has not been tested previously in the post-neoadjuvant setting [47]. In addition, clinical and pre-clinical data support deeper losses being associated with poorer outcome as reported in our study [33, 34]. Through RNA sequencing, we determined that deep losses of p53 are correlated with aberrant MYC-driven transcriptional amplification [35, 36]. These data are in accordance with work from others demonstrating that in MYC-driven cancers, p53 is a potent repressor of tumorigenesis, and losses in tumor suppressors such as p53 are likely required for supraphysiological MYC activity observed with focal amplification [48]. Certainly, while the loss of p53 is generally assumed to be a potent driver of TNBC, a graduated approach implicating increased risk-of-relapse and transcriptional amplification by MYC has not been previously presented. An exploratory analysis between CNV in RD samples and DFS revealed cancers bearing a copy gain of a region near SMAD4 on chromosome 18q were associated with poor prognosis, likely through upregulation of SMAD2. SMAD2 is a signal transducer of TGFß signaling, which has previously been implicated in the time-to-recurrence, chemoresistance, and metastatic capacity of TNBC [41, 49]. However, this particular genomic marker on 18q has not been previously reported as associated with recurrence.

Ultimately, the regulators we have identified in this chemorefractory population possess therapeutic potential. A number of clinical trials have been initiated in TNBC using immune checkpoint inhibitors with promising findings in the metastatic setting [50]. While we did not detect a robust association between immune infiltrate and recurrence in our dataset, due to the extreme depletion of immune activity that exists during chemotherapy, understanding how these drugs work in combination may be beneficial. In addition, a relatively small group of clinical trials investigating inhibitors of JAK-STAT, MEK-ERK, and TGFß have been initiated in TNBC. Our work supports innovative and expanded investigations using these targets and may also indicate the pre-clinical exploration of the interferon-λ due to its favorable selectivity and toxicity profile [15]. TP53 and MYC are considered seminal and undruggable drivers of TNBC. We have revealed evidence of strong synergy between these drivers in TNBC, and transcriptional amplification by their combined effort highlights pre-clinical work by others implicating RNA processing componentry such as the ribosome and spliceosome as synthetic lethal vulnerabilities in this aggressive subset [51, 52]. In addition, BET bromodomain inhibitors, which interfere with MYC-related transcription, are under investigation [53]. Interestingly, MYC amplification and MYC expression have been ruled out as biomarkers for response of bromodomain inhibition in TNBC, which, along with our work, warrants further exploration of p53 as a biomarker for this class of drugs [53, 54].

## Conclusions

Somatic aberrancy in chemorefractory TNBC genomes is dynamic during NAC, but largely with respect to CNV. However, individual trends in genomic evolution predictive of recurrence were not identified. During NAC, stem cell signaling is enriched and immune signaling is depleted, but it remains unclear what role these phenotypes play in relapse of TNBC. A powerful regulator of recurrence is somatic loss of TP53, for which a prognostically relevant continuum exists. Deep losses in TP53 predict inferior time-to-recurrence and result in transcriptional amplification by MYC, a therapeutic vulnerability. Additionally, somatic gains of a locus of 18q result in inferior disease-free survival associated with increased expression of SMAD2.

## Additional files

Additional file 1: **Figure S1.** Copy number heat map. Columns represent 75 individual patients across 70 genes in the targeted panel. Tumors are categorized by high-level amplification (red, 5.0+ copies), gain (yellow), normal (white), minor loss (royal blue), extensive loss (dark blue, < 0.5). (DOCX 679 kb)

Additional file 2: **Table S1.** Oncomine Cancer Panel target genes (PDF 170 kb)

Additional file 3: **Table S2.** RCB analysis (PDF 173 kb)

Additional file 4: **Table S3.** TCGA TNBCs copy number. CNVs called by GISTIC v2.0 across 135 TNBC tumors from the TCGA. (PDF 385 kb)

Additional file 5 : **Table S4.** 878 genes differentially expressed with ≤ 10%FDR between time points in matched pre- and post-chemotherapy tissues (*n* = 24 pairs). (PDF 217 kb)

Additional file 6: **Table S5.** Pre- and post-NAC upstream regulators (PDF 199 kb)

Additional file 7: **Table S6.** 508 genes with differential expression with ≤ 10%FDR between TP53 compound mutants and remaining tumors (*n* = 52). (PDF 202 kb)

Additional file 8: **Table S7.** Effectors of p53 inactivation upstream regulators (PDF 199 kb)

Additional file 9: **Table S8.** 18q21.1-21.2 expressed genes (PDF 186 kb)

Additional file 10: **Table S9.** Multivariate survival analysis statistics. *p* values are log-rank. (PDF 41 kb)

## Data Availability

The datasets used and/or analyzed during the current study are available from the corresponding author on reasonable request. Sequencing data will be submitted to the Gene Expression Omnibus database.
