# Supplementary material for: Profiling molecular regulators of recurrence in chemorefractory triple-negative breast cancers
Source: Breast Cancer Res. 2019 Aug 5;21:87. doi: 10.1186/s13058-019-1171-7 (PMC6683504; doi:10.1186/s13058-019-1171-7)

**Figure S1:** Copy number heat map. Columns represent 75 individual patients across 70 genes in the targeted panel. Tumors are categorized by high-level amplification (red, 5.0+ copies), gain (yellow), normal (white), minor loss (royal blue), extensive loss (dark blue, <0.5).


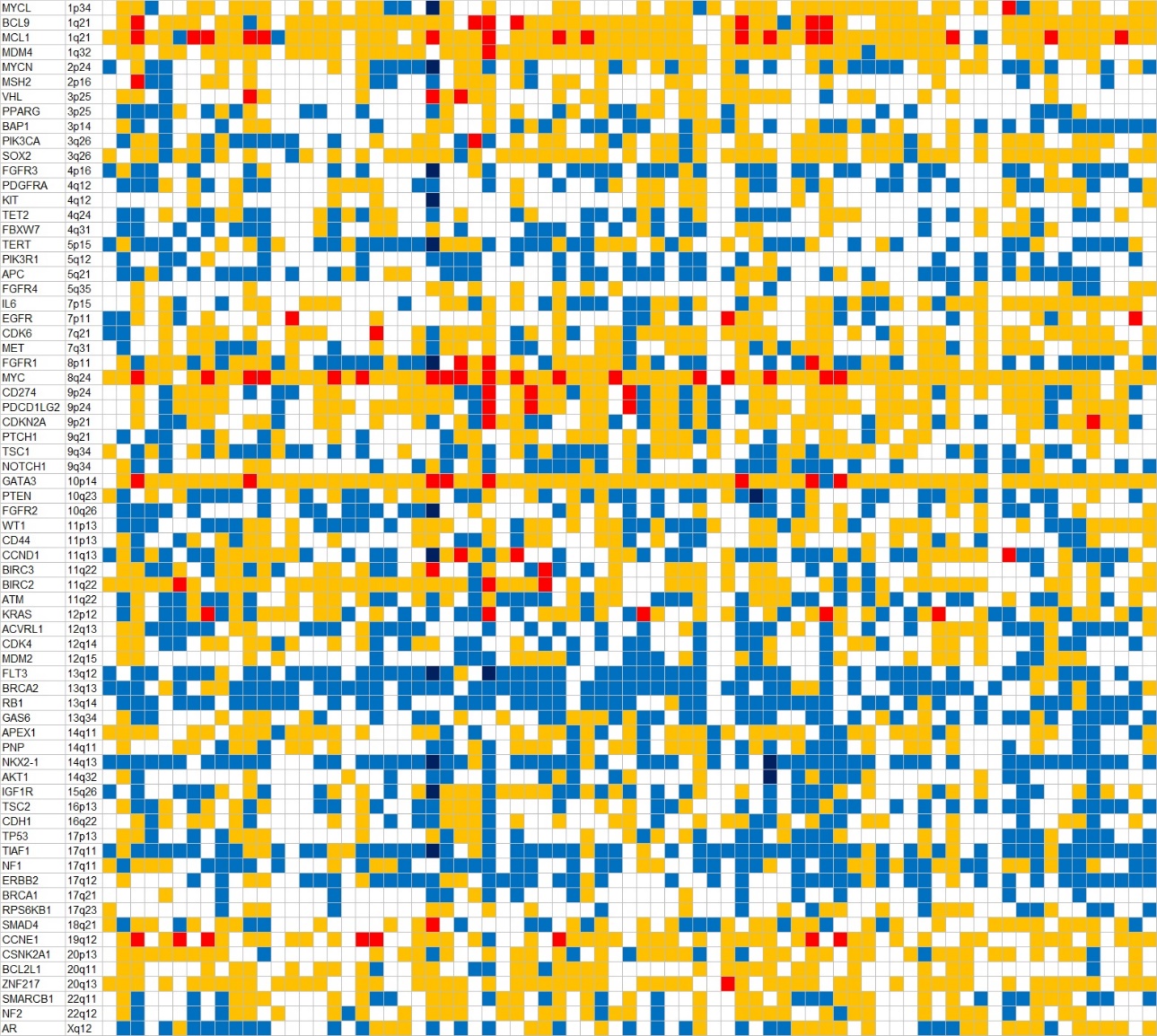

Supplement: Supplementary file 1 — Figure S1. Copy number heat map. Columns represent 75 individual patients across 70 genes in the targeted panel. Tumors are categorized by high-level amplification (red, 5.0+ copies), gain (yellow), normal (white), minor loss (royal blue), extensive loss (dark blue, < 0.5). (DOCX 679 kb) [file 13058_2019_1171_MOESM1_ESM.docx]
