# Supplementary material for: Profiling molecular regulators of recurrence in chemorefractory triple-negative breast cancers
Source: Breast Cancer Res. 2019 Aug 5;21:87. doi: 10.1186/s13058-019-1171-7 (PMC6683504; doi:10.1186/s13058-019-1171-7)
Supplement: Supplementary file 2 — Table S1. Oncomine Cancer Panel target genes (PDF 170 kb) [file 13058_2019_1171_MOESM2_ESM.pdf]

**Hotspot Genes**					
ABL1	CTNNB1	FOXL2	JAK2	MPL	RAF1
AKT1	DNMT3A	GATA2	JAK3	MTOR	RET
ALK	EGFR	GNA11	KDR	MYD88	RHEB
AR	ERBB2	GNAQ	KIT	NFE2L2	RHOA
BRAF	ERBB3	GNAS	KRAS	NRAS	SF3B1
BTK	ERBB4	HRAS	MAGOH	PAX5	SMO
C15orf23	ESR1	IDH1	MAP2K1	PDGFRA	SPOP
CBL	EZH2	IDH2	MAPK1	PIK3CA	SRC
CDK4	FGFR2	IFITM1	MAX	PPP2R1A	STAT3
CHEK2	FGFR3	IFITM3	MED12	PTPN11	U2AF1
CSF1R	FLT3	JAK1	MET	RAC1	XPO1
**Copy Number Variants**					
ACVRL1	CCND1	ERBB2	KIT	MYCN	TERT
AKT1	CCNE1	FGFR1	KRAS	NKX2-1	TIAF1
AR	CD274	FGFR2	MCL1	PDGFRA	ZNF217
APEX1	CD44	FGFR3	MDM2	PIK3CA	
BCL2L1	CDK4	FLT3	MDM4	PNP	
BCL9	CDK6	GAS6	MET	PPARG	
BIRC2	CSNK2A1	IGF1R	MYC	RPS6KB1	
BIRC3	EGFR	IL6	MYCL1	SOX2	
**Tumor Suppressors**					
APC	CDH1	NF1	PTEN	TP53	
ATM	CDKN2A	NF2	RB1	TSC1	
BAP1	FBXW7	NOTCH1	SMARCB1	TSC2	
BRCA1	GATA3	PIK3R1	SKT11	VHL	
BRCA2	MSH2	PTCH1	TET2	WT1	
