# Supplementary material for: Profiling molecular regulators of recurrence in chemorefractory triple-negative breast cancers
Source: Breast Cancer Res. 2019 Aug 5;21:87. doi: 10.1186/s13058-019-1171-7 (PMC6683504; doi:10.1186/s13058-019-1171-7)
Supplement: Supplementary file 3 — Table S2. RCB analysis (PDF 173 kb) [file 13058_2019_1171_MOESM3_ESM.pdf]

%Tumor	n	RCBI	RCBII	RCBIII
<60%	52	7 (13%)	36 (69%)	9 (17%)
≥60%	58	0 (0%)	26 (45%)	32 (55%)
