# Supplementary material for: Profiling molecular regulators of recurrence in chemorefractory triple-negative breast cancers
Source: Breast Cancer Res. 2019 Aug 5;21:87. doi: 10.1186/s13058-019-1171-7 (PMC6683504; doi:10.1186/s13058-019-1171-7)
Supplement: Supplementary file 4 — Table S3. TCGA TNBCs copy number. CNVs called by GISTIC v2.0 across 135 TNBC tumors from the TCGA. (PDF 385 kb) [file 13058_2019_1171_MOESM4_ESM.pdf]

TCGA Sample Number	MCL1	MYC	GATA3	RB1	BRCA2	TP53
TCGA-GM-A2DD-01	1	1	0	-1	-1	-1
TCGA-AO-A128-01	1	1	1	0	0	0
TCGA-E2-A14N-01	1	2	2	-1	1	0
TCGA-BH-A0RX-01	1	1	1	-1	-1	0
TCGA-A7-A5ZV-01	1	1	1	0	-1	0
TCGA-A2-A04T-01	2	2	1	1	2	-1
TCGA-BH-A1F6-01	1	1	1	-1	-1	-1
TCGA-E2-A1LI-01	1	1	1	0	0	-1
TCGA-A2-A0YE-01	1	0	1	-1	-1	-1
TCGA-BH-A18G-01	1	0	0	0	0	0
TCGA-A2-A0ST-01	1	0	1	0	0	0
TCGA-OL-A5RW-01	1	2	1	-1	0	-1
TCGA-A2-A0YM-01	1	1	1	-1	-1	0
TCGA-LL-A441-01	1	1	2	0	0	-1
TCGA-BH-A0B3-01	2	2	2	2	-1	-1
TCGA-A2-A0D0-01	2	2	1	-1	-1	-1
TCGA-A2-A04Q-01	1	2	1	-1	-1	0
TCGA-A8-A09X-01	0	1	1	-1	0	-1
TCGA-OL-A5D7-01	2	2	2	-1	-1	-1
TCGA-AO-A0J6-01	1	0	1	-1	-1	0
TCGA-AC-A2QH-01	0	2	-1	-1	-1	1
TCGA-D8-A147-01	2	1	0	-2	-2	-1
TCGA-E2-A1LL-01	0	-1	0	-1	-1	-1
TCGA-D8-A27F-01	0	2	0	-1	-1	0
TCGA-AR-A5QQ-01	0	1	-1	-1	-1	-1
TCGA-GM-A3XL-01	2	1	1	-1	0	-1
TCGA-C8-A12V-01	1	1	0	0	0	0
TCGA-A2-A0T0-01	1	2	2	0	0	-1
TCGA-B6-A1KF-01	2	1	2	-1	-1	0
TCGA-BH-A42U-01	0	0	0	0	0	0
TCGA-A8-A07O-01	2	2	0	-1	-1	-1
TCGA-AR-A1AI-01	1	1	1	-1	0	-1
TCGA-BH-A1FC-01	2	1	1	-2	-1	-1
TCGA-AR-A2LH-01	0	1	0	0	0	0
TCGA-C8-A134-01	0	2	-1	0	0	-1
TCGA-A2-A3XY-01	1	1	0	0	0	-1
TCGA-GM-A2DF-01	1	1	1	-1	-1	-1
TCGA-AN-A0AR-01	0	1	2	-1	1	0
TCGA-C8-A1HJ-01	1	1	1	-1	0	-1
TCGA-OL-A66P-01	0	1	0	0	0	0
TCGA-E2-A1LK-01	1	2	1	-1	-1	1
TCGA-BH-A1EW-01	1	1	1	-1	0	0
TCGA-A7-A26I-01	2	1	1	-1	-1	-1
TCGA-A7-A4SD-01	1	1	2	-1	-1	-1
TCGA-C8-A26Y-01	2	0	0	-1	-1	-1
TCGA-E2-A158-01	2	2	2	-1	-1	-1

TCGA-AO-A1KR-01	1	2	1	-1	-1	-1
TCGA-C8-A131-01	1	2	1	1	1	-1
TCGA-EW-A1PH-01	1	1	1	-1	-1	0
TCGA-A2-A0D2-01	1	1	1	-2	0	0
TCGA-BH-A0BL-01	2	1	2	1	-1	0
TCGA-A7-A0CE-01	2	1	0	-2	-2	0
TCGA-A7-A26G-01	0	1	2	-1	-1	-1
TCGA-E2-A150-01	1	2	1	-1	-1	-1
TCGA-AN-A0XU-01	1	2	2	2	0	-1
TCGA-BH-A0B9-01	1	0	0	-1	-1	-1
TCGA-GM-A2DI-01	0	0	0	0	0	-1
TCGA-AO-A0J4-01	2	2	0	-1	-1	-1
TCGA-AR-A1AY-01	1	2	1	-2	-1	-1
TCGA-A2-A3XT-01	1	1	1	-1	-1	-1
TCGA-E9-A5FL-01	0	2	0	1	1	-1
TCGA-AR-A0TS-01	1	2	1	0	0	0
TCGA-AR-A0U4-01	1	2	2	-1	-1	-1
TCGA-A2-A0T2-01	2	1	2	0	0	0
TCGA-AO-A124-01	1	1	1	1	-1	-1
TCGA-AR-A1AQ-01	1	1	2	0	0	-1
TCGA-A7-A0DA-01	2	2	2	1	1	-1
TCGA-EW-A1OW-01	1	1	1	-1	-1	0
TCGA-EW-A1P8-01	2	1	1	-1	-1	-1
TCGA-A1-A0SO-01	1	-1	1	1	1	-1
TCGA-D8-A1XQ-01	2	2	0	1	0	-1
TCGA-OL-A5D6-01	0	0	0	0	0	-1
TCGA-AN-A0AL-01	1	1	1	-1	0	-1
TCGA-D8-A27M-01	0	2	0	0	-1	-1
TCGA-BH-A0BG-01	1	2	1	0	0	1
TCGA-E2-A14R-01	2	2	1	-1	1	-1
TCGA-A8-A08R-01	1	2	1	0	0	-1
TCGA-D8-A1JF-01	1	1	1	1	1	1
TCGA-GM-A2DH-01	1	-1	1	-1	-1	-1
TCGA-A2-A3XS-01	2	1	1	0	0	0
TCGA-AN-A0AT-01	0	0	2	-1	0	-1
TCGA-A2-A04P-01	1	2	1	-1	1	-1
TCGA-AR-A0U1-01	1	2	0	-1	-1	-1
TCGA-E2-A1B6-01	0	1	0	0	0	-1
TCGA-AR-A1AR-01	2	0	1	-1	-1	-1
TCGA-C8-A27B-01	1	2	1	-1	-1	-1
TCGA-A2-A0SX-01	1	1	1	0	0	-1
TCGA-BH-A0E0-01	2	1	1	-1	-1	-1
TCGA-EW-A1P7-01	0	0	0	-2	-2	0
TCGA-EW-A1OV-01	1	0	1	-1	-1	-1
TCGA-AO-A129-01	2	2	2	-1	-1	-1
TCGA-D8-A13Z-01	1	0	1	-1	-1	-1
TCGA-AO-A0J2-01	1	0	1	0	1	-1

TCGA-D8-A143-01	1	1	0	-1	-1	1
TCGA-AR-A2LR-01	1	1	1	0	0	0
TCGA-E2-A1AZ-01	2	2	1	-1	-1	-1
TCGA-E2-A1LH-01	1	2	1	0	0	-1
TCGA-A7-A4SE-01	2	2	-1	0	-1	1
TCGA-D8-A1XK-01	1	1	2	-1	1	-1
TCGA-E2-A14X-01	1	1	-1	-1	1	-1
TCGA-EW-A1PB-01	1	2	1	0	0	0
TCGA-D8-A1JL-01	2	1	2	-1	1	0
TCGA-GM-A2DB-01	0	1	1	-1	-1	-1
TCGA-A2-A3XU-01	1	1	0	-1	0	1
TCGA-A7-A26F-01	1	1	2	1	1	-1
TCGA-AR-A256-01	2	2	1	0	-1	-1
TCGA-EW-A1P4-01	0	2	0	2	0	-1
TCGA-BH-A0WA-01	0	2	1	-1	-1	-1
TCGA-AN-A04D-01	1	1	1	-2	-2	-1
TCGA-D8-A142-01	1	1	1	-1	-1	0
TCGA-A1-A0SP-01	1	1	-1	-1	-1	-1
TCGA-AO-A12F-01	1	1	1	-1	1	0
TCGA-C8-A3M7-01	1	0	0	0	0	-1
TCGA-E2-A1LG-01	1	2	-1	-2	2	-1
TCGA-E2-A1L7-01	1	2	0	0	0	-1
TCGA-AQ-A54N-01	2	2	2	-1	-1	1
TCGA-A2-A3XX-01	1	1	1	-1	-1	1
TCGA-EW-A1P1-01	0	2	0	0	0	0
TCGA-A2-A0CM-01	2	2	2	-1	0	-1
TCGA-D8-A1JG-01	0	1	-1	-1	-1	-1
TCGA-A2-A1G6-01	0	0	0	0	0	0
TCGA-AC-A2QJ-01	1	1	-1	-1	-1	-1
TCGA-OL-A66I-01	2	2	1	-1	-1	-1
TCGA-E2-A573-01	1	1	1	0	0	-1
TCGA-AC-A2BK-01	2	1	1	-1	-1	0
TCGA-EW-A3U0-01	1	2	1	-1	-1	2
TCGA-GI-A2C9-01	1	1	1	0	0	-1
TCGA-C8-A26X-01	1	1	1	-1	-1	-1
TCGA-D8-A27H-01	0	2	-1	0	0	-1
TCGA-A1-A0SK-01	1	1	-1	-2	1	-1
TCGA-C8-A12L-01	1	1	1	1	-1	-1
TCGA-BH-A0E6-01	1	0	1	-2	0	-1
TCGA-AQ-A04J-01	2	2	0	-2	0	-1
TCGA-AO-A03U-01	0	0	-1	1	1	0
TCGA-BH-A18V-01	2	2	1	1	1	-1

TCGA	MCL1	MYC	GATA3	RB1	BRCA2	TP53
Amp	25%	37%	17%	2%	1%	1%
Gain	56%	47%	51%	10%	13%	7%
Normal	19%	14%	23%	36%	36%	26%
Loss	0%	2%	9%	61%	50%	67%
BRE09-146						
Amp	21%	24%	11%	0%	0%	0%
Gain	75%	77%	88%	4%	11%	24%
Normal	9%	4%	3%	33%	23%	49%
Loss	4%	0%	1%	63%	67%	27%
