# Supplementary material for: Profiling molecular regulators of recurrence in chemorefractory triple-negative breast cancers
Source: Breast Cancer Res. 2019 Aug 5;21:87. doi: 10.1186/s13058-019-1171-7 (PMC6683504; doi:10.1186/s13058-019-1171-7)
Supplement: Supplementary file 5 — : Table S4. 878 genes differentially expressed with ≤ 10%FDR between time points in matched pre- and post-chemotherapy tissues (n = 24 pairs). (PDF 217 kb) [file 13058_2019_1171_MOESM5_ESM.pdf]

Gene Symbol	p-value(Post- vs. Pre-chemotherapy)	Fold-Change(Post- vs. Pre-chemotherapy)	Fold-Change(Post- vs. Pre-chemotherapy) (Description)
FOSB	2.91E-06	15.1637	Post- up vs Pre-chemotherapy
ZNF778	4.85E-06	-7.41356	Post- down vs Pre-chemotherapy
SCAPER	6.43E-06	-7.01268	Post- down vs Pre-chemotherapy
TXNRD3	1.39E-05	-6.25876	Post- down vs Pre-chemotherapy
METTL3	1.83E-05	-12.2916	Post- down vs Pre-chemotherapy
TBRG1	1.94E-05	-3.53897	Post- down vs Pre-chemotherapy
KMT2A	3.19E-05	-2.21327	Post- down vs Pre-chemotherapy
WSB2	3.33E-05	-3.51436	Post- down vs Pre-chemotherapy
IL16	3.73E-05	-3.76154	Post- down vs Pre-chemotherapy
ZNF557	3.93E-05	-3.90347	Post- down vs Pre-chemotherapy
SLC15A2	4.34E-05	-8.42846	Post- down vs Pre-chemotherapy
PRKAG2	6.97E-05	-2.3864	Post- down vs Pre-chemotherapy
FAM46C	7.66E-05	-8.37912	Post- down vs Pre-chemotherapy
FCGR3A	7.76E-05	-3.70668	Post- down vs Pre-chemotherapy
ZNF430	8.23E-05	-7.60874	Post- down vs Pre-chemotherapy
NFYB	8.62E-05	-5.7199	Post- down vs Pre-chemotherapy
FOS	9.12E-05	15.0827	Post- up vs Pre-chemotherapy
FAM134C	9.37E-05	-2.72585	Post- down vs Pre-chemotherapy
KCNA3	9.57E-05	-4.91196	Post- down vs Pre-chemotherapy
NOD1	0.000112243	-5.18271	Post- down vs Pre-chemotherapy
TOLLIP	0.000121139	-4.67391	Post- down vs Pre-chemotherapy
PHACTR4	0.000140658	-2.92924	Post- down vs Pre-chemotherapy
VPS13A	0.000158696	-3.09905	Post- down vs Pre-chemotherapy
PARP9	0.000163167	-2.71441	Post- down vs Pre-chemotherapy
MIR409	0.000163428	22.8896	Post- up vs Pre-chemotherapy
CXCL11	0.000169303	-16.4714	Post- down vs Pre-chemotherapy
CTTNBP2NL	0.00017103	-3.24683	Post- down vs Pre-chemotherapy
CXCL13	0.00017528	-15.8362	Post- down vs Pre-chemotherapy
PEX11G	0.000178047	3.255	Post- up vs Pre-chemotherapy
AP1G1	0.000183346	-2.59549	Post- down vs Pre-chemotherapy
IL21R	0.000191112	-5.48694	Post- down vs Pre-chemotherapy
SGCE	0.000193613	-6.37238	Post- down vs Pre-chemotherapy
LOC613037	0.000194445	-6.6593	Post- down vs Pre-chemotherapy
ZBTB34	0.000210331	-4.15114	Post- down vs Pre-chemotherapy
GPR161	0.000215305	-2.79672	Post- down vs Pre-chemotherapy
ULK4	0.000219479	-4.23	Post- down vs Pre-chemotherapy
QRSL1	0.000236322	-4.07297	Post- down vs Pre-chemotherapy
ZNF354B	0.000237956	-7.35054	Post- down vs Pre-chemotherapy
FANCF	0.000238205	-4.63794	Post- down vs Pre-chemotherapy
SMG1	0.000245981	-2.05889	Post- down vs Pre-chemotherapy
THEM4	0.000246436	-6.99939	Post- down vs Pre-chemotherapy
ZNF292	0.000258507	-2.17769	Post- down vs Pre-chemotherapy
MPHOSPH8	0.000261688	-3.70434	Post- down vs Pre-chemotherapy
ACO2	0.000263156	-4.13232	Post- down vs Pre-chemotherapy
AGA	0.000277202	-7.46944	Post- down vs Pre-chemotherapy
MMP11	0.00029085	-4.73659	Post- down vs Pre-chemotherapy
MIR27A	0.000294354	3.12611	Post- up vs Pre-chemotherapy
PAX8-AS1	0.000304586	-4.20268	Post- down vs Pre-chemotherapy
DOCK2	0.000308777	-4.64971	Post- down vs Pre-chemotherapy
MIR4521	0.000320167	-44.4529	Post- down vs Pre-chemotherapy
METTL4	0.000336049	-4.16601	Post- down vs Pre-chemotherapy
MIR125B2	0.000340447	3.82056	Post- up vs Pre-chemotherapy
GOT1	0.000341225	-7.29639	Post- down vs Pre-chemotherapy
RABGGTB	0.000344718	-6.87114	Post- down vs Pre-chemotherapy
MIR4484	0.000353994	11.097	Post- up vs Pre-chemotherapy
SLC30A1	0.000356491	-3.5521	Post- down vs Pre-chemotherapy
TPR	0.000368729	-2.96333	Post- down vs Pre-chemotherapy
CCNG2	0.000383779	-2.1006	Post- down vs Pre-chemotherapy
C11orf71	0.000394467	-4.74521	Post- down vs Pre-chemotherapy
SRBD1	0.000409951	-4.86187	Post- down vs Pre-chemotherapy
DDX39B	0.000413054	-2.46673	Post- down vs Pre-chemotherapy
PISD	0.000413261	-3.42565	Post- down vs Pre-chemotherapy
NBPF10	0.000422187	-3.78656	Post- down vs Pre-chemotherapy
WNT5A	0.000424309	-3.16204	Post- down vs Pre-chemotherapy
INPP5D	0.000432147	-3.32624	Post- down vs Pre-chemotherapy
LOC100862671	0.000434372	-6.28565	Post- down vs Pre-chemotherapy
ZNF137P	0.000440222	-6.18017	Post- down vs Pre-chemotherapy
CHUK	0.000445081	-6.11993	Post- down vs Pre-chemotherapy
EDEM1	0.000490507	-2.08099	Post- down vs Pre-chemotherapy
FASTKD3	0.00049151	-6.31981	Post- down vs Pre-chemotherapy
NR2C1	0.000493242	-5.95805	Post- down vs Pre-chemotherapy

CD58	0.000495813	-5.73124	Post- down vs Pre-chemotherapy
SLFN13	0.000509992	-3.97118	Post- down vs Pre-chemotherapy
AP3S1	0.000511267	-9.31361	Post- down vs Pre-chemotherapy
LYSMD3	0.000517079	-3.23061	Post- down vs Pre-chemotherapy
DPH3	0.000539666	-6.65177	Post- down vs Pre-chemotherapy
SMG1P2	0.000551654	-4.79258	Post- down vs Pre-chemotherapy
NDUFC1	0.000554203	-5.60391	Post- down vs Pre-chemotherapy
ZNF766	0.000562824	-4.47509	Post- down vs Pre-chemotherapy
WDR5B	0.00057781	-4.26802	Post- down vs Pre-chemotherapy
ZNF765	0.000581699	-4.76888	Post- down vs Pre-chemotherapy
MINOS1	0.000586466	-3.76009	Post- down vs Pre-chemotherapy
CNKSR1	0.000593639	-6.73277	Post- down vs Pre-chemotherapy
NEBL	0.00060771	-4.2308	Post- down vs Pre-chemotherapy
LRRC37A4P	0.000610648	-3.72487	Post- down vs Pre-chemotherapy
KAZN	0.000616209	-2.0389	Post- down vs Pre-chemotherapy
SZT2	0.000649664	-2.12709	Post- down vs Pre-chemotherapy
TRIM25	0.000652064	-3.31202	Post- down vs Pre-chemotherapy
MIR92A1	0.000660321	3.9366	Post- up vs Pre-chemotherapy
ORMDL3	0.000663415	-2.64981	Post- down vs Pre-chemotherapy
SLC22A5	0.000664503	-4.5669	Post- down vs Pre-chemotherapy
CCDC84	0.000670306	-4.65057	Post- down vs Pre-chemotherapy
DUSP1	0.000673538	7.28153	Post- up vs Pre-chemotherapy
FLNB	0.000685259	-2.08184	Post- down vs Pre-chemotherapy
PTPRCAP	0.000690006	-5.65472	Post- down vs Pre-chemotherapy
UBE2DNL	0.000691734	3.40063	Post- up vs Pre-chemotherapy
PHF14	0.000698177	-5.50323	Post- down vs Pre-chemotherapy
ZMYM5	0.000715774	-4.54673	Post- down vs Pre-chemotherapy
FBXO48	0.000719574	-5.04	Post- down vs Pre-chemotherapy
OXSR1	0.000720464	-2.74387	Post- down vs Pre-chemotherapy
BLCAP	0.000722365	-2.21601	Post- down vs Pre-chemotherapy
IKZF3	0.00072736	-3.5732	Post- down vs Pre-chemotherapy
PAG1	0.000737857	-3.35977	Post- down vs Pre-chemotherapy
DNMT3A	0.000739903	-2.81028	Post- down vs Pre-chemotherapy
DDX23	0.00075203	-2.21484	Post- down vs Pre-chemotherapy
SYTL3	0.000757698	-5.86153	Post- down vs Pre-chemotherapy
MIR143	0.000761961	4.10393	Post- up vs Pre-chemotherapy
CTAGE5	0.000764829	-5.58692	Post- down vs Pre-chemotherapy
PRKAG1	0.000766037	-5.27183	Post- down vs Pre-chemotherapy
CWF19L2	0.000768253	-4.98312	Post- down vs Pre-chemotherapy
HLA-DQA1	0.00079081	-5.42508	Post- down vs Pre-chemotherapy
MBD2	0.000794858	-2.23058	Post- down vs Pre-chemotherapy
CCP110	0.000796274	-3.25042	Post- down vs Pre-chemotherapy
CEP162	0.000808144	-4.01181	Post- down vs Pre-chemotherapy
MTMR4	0.000812402	-3.39702	Post- down vs Pre-chemotherapy
BCKDHA	0.000843462	-2.6487	Post- down vs Pre-chemotherapy
LCOR	0.000851121	-2.35018	Post- down vs Pre-chemotherapy
ZXDC	0.000851704	-2.36113	Post- down vs Pre-chemotherapy
ANKZF1	0.000852141	-3.09236	Post- down vs Pre-chemotherapy
METTL6	0.000864688	-3.1625	Post- down vs Pre-chemotherapy
MIR320A	0.000867989	2.20698	Post- up vs Pre-chemotherapy
CALU	0.000881063	-2.79399	Post- down vs Pre-chemotherapy
NEDD4L	0.000915185	-3.0821	Post- down vs Pre-chemotherapy
DOCK10	0.000928072	-2.71054	Post- down vs Pre-chemotherapy
PSRC1	0.00093621	-9.53289	Post- down vs Pre-chemotherapy
LSM8	0.000950824	-2.09524	Post- down vs Pre-chemotherapy
RAPGEF2	0.00098914	-2.33526	Post- down vs Pre-chemotherapy
PKP4	0.000990501	-2.74869	Post- down vs Pre-chemotherapy
PURA	0.000991865	-2.5569	Post- down vs Pre-chemotherapy
ARL5A	0.000994247	-3.51549	Post- down vs Pre-chemotherapy
CCDC14	0.000995892	-3.16863	Post- down vs Pre-chemotherapy
IFIH1	0.00100143	-4.03701	Post- down vs Pre-chemotherapy
EMC3-AS1	0.00103	-3.35977	Post- down vs Pre-chemotherapy
AGPAT3	0.00104133	-2.27659	Post- down vs Pre-chemotherapy
MPHOSPH9	0.00104452	-4.16549	Post- down vs Pre-chemotherapy
ROR1	0.00104514	-3.4219	Post- down vs Pre-chemotherapy
AACS	0.00105153	-3.71161	Post- down vs Pre-chemotherapy
R3HCC1L	0.00106654	-4.14472	Post- down vs Pre-chemotherapy
KIF13A	0.00106793	-2.46506	Post- down vs Pre-chemotherapy
PRKX	0.00107475	-4.43614	Post- down vs Pre-chemotherapy
H2AFJ	0.00107632	-2.73569	Post- down vs Pre-chemotherapy
FRYL	0.00108517	-3.23633	Post- down vs Pre-chemotherapy
ISLR	0.00109626	-2.53887	Post- down vs Pre-chemotherapy
RABGAP1	0.00110747	-2.91496	Post- down vs Pre-chemotherapy

SDCCAG3	0.00110747	-2.48311	Post- down vs Pre-chemotherapy
ACSL5	0.00111028	-4.69539	Post- down vs Pre-chemotherapy
TCP11L1	0.00111149	-4.0815	Post- down vs Pre-chemotherapy
TEP1	0.00111368	-3.70753	Post- down vs Pre-chemotherapy
STK17A	0.00112206	-4.01657	Post- down vs Pre-chemotherapy
ARL6IP5	0.00112521	-3.21656	Post- down vs Pre-chemotherapy
ANO8	0.00112639	-3.03768	Post- down vs Pre-chemotherapy
ILKAP	0.00112942	-5.08697	Post- down vs Pre-chemotherapy
LRRFIP2	0.00113054	-3.70723	Post- down vs Pre-chemotherapy
ZNF350	0.00113479	-6.69696	Post- down vs Pre-chemotherapy
FAT1	0.00114306	-2.18645	Post- down vs Pre-chemotherapy
MRAS	0.00114885	-3.08364	Post- down vs Pre-chemotherapy
NBEAL2	0.00114904	-2.25223	Post- down vs Pre-chemotherapy
FAN1	0.00115429	-2.49339	Post- down vs Pre-chemotherapy
ASCC3	0.00116701	-2.42087	Post- down vs Pre-chemotherapy
NIPA1	0.00117657	-2.6035	Post- down vs Pre-chemotherapy
SLA	0.00119022	-3.15446	Post- down vs Pre-chemotherapy
PRR14	0.00119185	-3.01362	Post- down vs Pre-chemotherapy
CD276	0.00119904	-3.48887	Post- down vs Pre-chemotherapy
CLEC2D	0.00120692	-3.67024	Post- down vs Pre-chemotherapy
WHAMMP1	0.00120958	-5.68494	Post- down vs Pre-chemotherapy
LSM3	0.00121486	-8.72404	Post- down vs Pre-chemotherapy
ZNF730	0.00121794	-4.63943	Post- down vs Pre-chemotherapy
KATNBL1	0.0012201	-5.28975	Post- down vs Pre-chemotherapy
SLC35C1	0.00122151	-2.71224	Post- down vs Pre-chemotherapy
PRPSAP2	0.00122869	-3.36695	Post- down vs Pre-chemotherapy
ZNF552	0.00122943	-5.70746	Post- down vs Pre-chemotherapy
LACC1	0.00123111	-5.51126	Post- down vs Pre-chemotherapy
SLC7A11	0.00123576	-2.94633	Post- down vs Pre-chemotherapy
PGRMC2	0.00124486	-2.74795	Post- down vs Pre-chemotherapy
MIR136	0.00125337	6.52223	Post- up vs Pre-chemotherapy
STX2	0.00125396	-3.78506	Post- down vs Pre-chemotherapy
B4GALT4	0.00125914	-4.12692	Post- down vs Pre-chemotherapy
ARL3	0.00126515	-3.86677	Post- down vs Pre-chemotherapy
BTN2A1	0.00127656	-3.64862	Post- down vs Pre-chemotherapy
SUGP1	0.00128478	-3.11367	Post- down vs Pre-chemotherapy
PLA2R1	0.00130317	-2.46322	Post- down vs Pre-chemotherapy
ZKSCAN1	0.00131031	-2.77431	Post- down vs Pre-chemotherapy
ZNF726	0.00131386	-5.14098	Post- down vs Pre-chemotherapy
ADAM1A	0.00133822	-4.0696	Post- down vs Pre-chemotherapy
POLG	0.00133982	-3.31607	Post- down vs Pre-chemotherapy
GPR107	0.00134933	-3.65518	Post- down vs Pre-chemotherapy
FAM26F	0.00136107	-4.64	Post- down vs Pre-chemotherapy
NXF1	0.00137117	-3.90338	Post- down vs Pre-chemotherapy
HAUS3	0.00137429	-3.16143	Post- down vs Pre-chemotherapy
SYS1	0.00137741	-2.82945	Post- down vs Pre-chemotherapy
ZNF160	0.00138382	-4.47002	Post- down vs Pre-chemotherapy
USP4	0.00139201	-2.41451	Post- down vs Pre-chemotherapy
ZNF207	0.00139325	-3.32599	Post- down vs Pre-chemotherapy
PCNXL4	0.0013937	-2.36241	Post- down vs Pre-chemotherapy
MIR376C	0.0013998	11.0885	Post- up vs Pre-chemotherapy
ZNF8	0.00141947	-3.87565	Post- down vs Pre-chemotherapy
NASP	0.0014225	-2.89131	Post- down vs Pre-chemotherapy
ZNF528	0.00144044	-4.10024	Post- down vs Pre-chemotherapy
SAMD9L	0.00144098	-4.90796	Post- down vs Pre-chemotherapy
NBAS	0.00147612	-3.20709	Post- down vs Pre-chemotherapy
BLZF1	0.00148013	-4.02101	Post- down vs Pre-chemotherapy
ANKAR	0.00148792	-4.01645	Post- down vs Pre-chemotherapy
SNRNP35	0.00148845	-3.87806	Post- down vs Pre-chemotherapy
ARMCX3	0.0015091	-3.87795	Post- down vs Pre-chemotherapy
MANF	0.00152937	-5.29225	Post- down vs Pre-chemotherapy
COPS8	0.00152942	-5.04454	Post- down vs Pre-chemotherapy
MRPL17	0.0015326	-2.42611	Post- down vs Pre-chemotherapy
TTC13	0.00154486	-3.32013	Post- down vs Pre-chemotherapy
DIS3L2	0.00156323	-3.45737	Post- down vs Pre-chemotherapy
ERGIC2	0.00156794	-2.48226	Post- down vs Pre-chemotherapy
CEP95	0.00156924	-6.39853	Post- down vs Pre-chemotherapy
GBP1	0.00157206	-3.47489	Post- down vs Pre-chemotherapy
PKNOX1	0.00158472	-2.99497	Post- down vs Pre-chemotherapy
CEP70	0.0015992	-3.82038	Post- down vs Pre-chemotherapy
SLC1A4	0.00160897	-3.06304	Post- down vs Pre-chemotherapy
VPS37A	0.00160924	-3.14682	Post- down vs Pre-chemotherapy
LOC101926933	0.00161209	-7.32433	Post- down vs Pre-chemotherapy

ZNF506	0.00161522	-4.66629	Post- down vs Pre-chemotherapy
CD180	0.00162562	-3.35322	Post- down vs Pre-chemotherapy
HLA-DQB1	0.00163075	-4.53428	Post- down vs Pre-chemotherapy
HCFC2	0.00163956	-3.20708	Post- down vs Pre-chemotherapy
CYBB	0.00164368	-3.97604	Post- down vs Pre-chemotherapy
EIF4EBP1	0.00165876	-5.88914	Post- down vs Pre-chemotherapy
PTCD3	0.0016618	-2.77601	Post- down vs Pre-chemotherapy
PLEKHA3	0.00167227	-2.67176	Post- down vs Pre-chemotherapy
CEP97	0.00167354	-3.69959	Post- down vs Pre-chemotherapy
ELF2	0.0016738	-4.08019	Post- down vs Pre-chemotherapy
CLCN6	0.00168085	-2.63488	Post- down vs Pre-chemotherapy
FAM111B	0.00168322	-3.55968	Post- down vs Pre-chemotherapy
RBM23	0.00168758	-3.05016	Post- down vs Pre-chemotherapy
C16orf72	0.00169968	-3.79619	Post- down vs Pre-chemotherapy
LIMK2	0.00169995	-3.2347	Post- down vs Pre-chemotherapy
HIST1H3G	0.00170204	-7.65429	Post- down vs Pre-chemotherapy
KCTD7	0.00170662	-3.0703	Post- down vs Pre-chemotherapy
RNF213	0.00171365	-2.32519	Post- down vs Pre-chemotherapy
TSPAN2	0.00171678	-3.6594	Post- down vs Pre-chemotherapy
CASP3	0.00171825	-4.3109	Post- down vs Pre-chemotherapy
CHD9	0.00172346	-2.58471	Post- down vs Pre-chemotherapy
ARHGEF3	0.00172504	-3.22732	Post- down vs Pre-chemotherapy
ZKSCAN8	0.0017281	-2.82045	Post- down vs Pre-chemotherapy
RBFA	0.00172862	-3.45579	Post- down vs Pre-chemotherapy
CASP8AP2	0.00174519	-2.72486	Post- down vs Pre-chemotherapy
FOXF2	0.00174734	4.88666	Post- up vs Pre-chemotherapy
ZNF253	0.00174755	-4.60756	Post- down vs Pre-chemotherapy
MIR17HG	0.00176132	-2.24777	Post- down vs Pre-chemotherapy
SLC25A37	0.00176264	-4.32809	Post- down vs Pre-chemotherapy
ZNF136	0.00177023	-3.74323	Post- down vs Pre-chemotherapy
SMG5	0.00178553	-2.13002	Post- down vs Pre-chemotherapy
ZEB2	0.00179358	-2.10547	Post- down vs Pre-chemotherapy
GOT2	0.00181742	-4.6706	Post- down vs Pre-chemotherapy
IFNAR2	0.00181831	-2.52755	Post- down vs Pre-chemotherapy
HECTD1	0.00183146	-2.13101	Post- down vs Pre-chemotherapy
COL12A1	0.00183201	-2.01732	Post- down vs Pre-chemotherapy
POFUT2	0.00183821	-2.49237	Post- down vs Pre-chemotherapy
ZNF433	0.00185609	-4.4974	Post- down vs Pre-chemotherapy
RELT	0.00185658	-3.452	Post- down vs Pre-chemotherapy
EMC8	0.00185777	-3.55851	Post- down vs Pre-chemotherapy
USO1	0.00186288	-2.25742	Post- down vs Pre-chemotherapy
ZNF614	0.00186316	-4.10413	Post- down vs Pre-chemotherapy
ZNF680	0.00186681	-4.09228	Post- down vs Pre-chemotherapy
MIR140	0.00186987	2.94696	Post- up vs Pre-chemotherapy
PIGX	0.00187721	-3.09178	Post- down vs Pre-chemotherapy
ZNF710	0.00188288	-3.2825	Post- down vs Pre-chemotherapy
MIR542	0.00188371	7.80546	Post- up vs Pre-chemotherapy
TVP23C	0.00189502	-3.14149	Post- down vs Pre-chemotherapy
RSAD2	0.00189698	-3.74453	Post- down vs Pre-chemotherapy
STRIP1	0.00191467	-3.07836	Post- down vs Pre-chemotherapy
LNX2	0.00191509	-3.70189	Post- down vs Pre-chemotherapy
SLC8A1	0.00192949	-2.90361	Post- down vs Pre-chemotherapy
PDPR	0.00192972	-3.17355	Post- down vs Pre-chemotherapy
TRIM13	0.00193004	-3.94755	Post- down vs Pre-chemotherapy
CDK2AP1	0.00193628	-2.17776	Post- down vs Pre-chemotherapy
NBPF25P	0.00194079	-4.99069	Post- down vs Pre-chemotherapy
WIPI1	0.00195007	-4.433	Post- down vs Pre-chemotherapy
ZNF675	0.00196599	-4.50991	Post- down vs Pre-chemotherapy
MIS12	0.0019749	-3.43632	Post- down vs Pre-chemotherapy
SF3A3	0.00197598	-2.76411	Post- down vs Pre-chemotherapy
DNAJC16	0.00198221	-3.58841	Post- down vs Pre-chemotherapy
CEP128	0.00198467	-3.57667	Post- down vs Pre-chemotherapy
AGAP9	0.00198709	-4.94505	Post- down vs Pre-chemotherapy
UBTD2	0.00199058	-2.57923	Post- down vs Pre-chemotherapy
ZNF182	0.00201264	-5.4045	Post- down vs Pre-chemotherapy
CYB561	0.00201408	-2.91725	Post- down vs Pre-chemotherapy
ZCCHC6	0.00201409	-3.1055	Post- down vs Pre-chemotherapy
TSPAN5	0.00202266	-2.69171	Post- down vs Pre-chemotherapy
SNX6	0.00202321	-3.05318	Post- down vs Pre-chemotherapy
STAT1	0.00202504	-2.4488	Post- down vs Pre-chemotherapy
FYTTD1	0.00202572	-3.37699	Post- down vs Pre-chemotherapy
VDAC2	0.00202775	-3.2828	Post- down vs Pre-chemotherapy
RAB7L1	0.00203152	-3.18314	Post- down vs Pre-chemotherapy

SLC6A9	0.00204826	-3.15837	Post- down vs Pre-chemotherapy
GNL2	0.00206629	-4.18078	Post- down vs Pre-chemotherapy
ITGA4	0.00208598	-2.68181	Post- down vs Pre-chemotherapy
CAP1	0.00210831	-2.17767	Post- down vs Pre-chemotherapy
MTG1	0.00211199	-5.59638	Post- down vs Pre-chemotherapy
S100PBP	0.00211689	-3.48448	Post- down vs Pre-chemotherapy
ARHGAP9	0.00212498	-2.66282	Post- down vs Pre-chemotherapy
LRBA	0.00213404	-2.62445	Post- down vs Pre-chemotherapy
TBCK	0.00214545	-2.80367	Post- down vs Pre-chemotherapy
UBE3B	0.00216447	-2.35679	Post- down vs Pre-chemotherapy
STK36	0.00216484	-3.28809	Post- down vs Pre-chemotherapy
STXBP4	0.00216785	-3.36046	Post- down vs Pre-chemotherapy
TBCCD1	0.00217001	-4.29655	Post- down vs Pre-chemotherapy
NAA30	0.00218523	-3.24742	Post- down vs Pre-chemotherapy
QSER1	0.00218621	-3.63494	Post- down vs Pre-chemotherapy
SAFB2	0.00219067	-2.48025	Post- down vs Pre-chemotherapy
UBAP2	0.00220384	-2.6823	Post- down vs Pre-chemotherapy
CFDP1	0.00222267	-5.11227	Post- down vs Pre-chemotherapy
SLC12A6	0.00222868	-2.43847	Post- down vs Pre-chemotherapy
FAM127A	0.00222922	-3.26868	Post- down vs Pre-chemotherapy
OFD1	0.00224338	-4.3943	Post- down vs Pre-chemotherapy
ADCK4	0.00225522	-3.76723	Post- down vs Pre-chemotherapy
PITPNM2	0.00226446	-3.46942	Post- down vs Pre-chemotherapy
CLN3	0.00229361	-3.03578	Post- down vs Pre-chemotherapy
MRPS6	0.00230066	-7.27415	Post- down vs Pre-chemotherapy
GPHN	0.00230492	-2.80666	Post- down vs Pre-chemotherapy
SEMA4D	0.00231788	-2.76542	Post- down vs Pre-chemotherapy
DGCR11	0.00232922	-3.68753	Post- down vs Pre-chemotherapy
ORC4	0.00232983	-2.90595	Post- down vs Pre-chemotherapy
ATMIN	0.0023411	-2.03471	Post- down vs Pre-chemotherapy
RASSF3	0.00234286	-4.08629	Post- down vs Pre-chemotherapy
SUCLG1	0.00234651	-6.04302	Post- down vs Pre-chemotherapy
RNF10	0.00235404	-2.59446	Post- down vs Pre-chemotherapy
PLXNA3	0.0023547	-2.32529	Post- down vs Pre-chemotherapy
TAS2R13	0.00236673	-4.28181	Post- down vs Pre-chemotherapy
MIR339	0.00236856	3.98247	Post- up vs Pre-chemotherapy
PSMD6	0.00237286	-3.04831	Post- down vs Pre-chemotherapy
C3orf17	0.0024044	-2.93344	Post- down vs Pre-chemotherapy
TNFAIP8	0.00241196	-2.88766	Post- down vs Pre-chemotherapy
MCF2L	0.00241418	-3.33232	Post- down vs Pre-chemotherapy
NOC3L	0.00241786	-4.14525	Post- down vs Pre-chemotherapy
CEPT1	0.00242161	-3.36989	Post- down vs Pre-chemotherapy
FBXO22	0.00243081	-4.17398	Post- down vs Pre-chemotherapy
ISCU	0.0024381	-4.29845	Post- down vs Pre-chemotherapy
MIR1247	0.00244497	5.11807	Post- up vs Pre-chemotherapy
SULF1	0.00245099	-2.38718	Post- down vs Pre-chemotherapy
NR1H3	0.00245799	-3.13393	Post- down vs Pre-chemotherapy
MROH1	0.00245834	-2.20589	Post- down vs Pre-chemotherapy
SNX8	0.00246758	-3.72933	Post- down vs Pre-chemotherapy
LIG1	0.00246857	-2.3045	Post- down vs Pre-chemotherapy
NADSYN1	0.00247538	-4.65634	Post- down vs Pre-chemotherapy
ANKRD36	0.0024856	-3.47039	Post- down vs Pre-chemotherapy
DYRK1A	0.00249097	-2.52351	Post- down vs Pre-chemotherapy
ZNF800	0.0024957	-2.88968	Post- down vs Pre-chemotherapy
MIRLET7C	0.00251123	3.13407	Post- up vs Pre-chemotherapy
ZNF335	0.0025172	-2.61678	Post- down vs Pre-chemotherapy
KNTC1	0.00252484	-2.91595	Post- down vs Pre-chemotherapy
SYNJ1	0.00254737	-2.24329	Post- down vs Pre-chemotherapy
PCDH17	0.00254754	-2.77243	Post- down vs Pre-chemotherapy
MSANTD2	0.00254777	-3.5357	Post- down vs Pre-chemotherapy
KANSL1	0.00255897	-2.0262	Post- down vs Pre-chemotherapy
MTBP	0.00256121	-2.41389	Post- down vs Pre-chemotherapy
STARD3NL	0.00258483	-4.09329	Post- down vs Pre-chemotherapy
PARP14	0.00259292	-2.99044	Post- down vs Pre-chemotherapy
SNORD113-6	0.00259544	2.436	Post- up vs Pre-chemotherapy
ARHGAP25	0.00259726	-3.02866	Post- down vs Pre-chemotherapy
NKIRAS1	0.00260283	-5.8097	Post- down vs Pre-chemotherapy
DIAPH2	0.00260489	-3.01237	Post- down vs Pre-chemotherapy
TSG101	0.00262444	-6.69813	Post- down vs Pre-chemotherapy
CASC5	0.00264538	-3.26573	Post- down vs Pre-chemotherapy
PDCD11	0.00264833	-2.81689	Post- down vs Pre-chemotherapy
FANCD2	0.00267593	-2.95459	Post- down vs Pre-chemotherapy
PREX1	0.00267714	-3.2437	Post- down vs Pre-chemotherapy

ABHD17B	0.00268908	-2.94023	Post- down vs Pre-chemotherapy
ZC3HAV1	0.00269896	-2.84875	Post- down vs Pre-chemotherapy
TAF7	0.00269988	-2.62741	Post- down vs Pre-chemotherapy
CDK9	0.00270414	-3.1787	Post- down vs Pre-chemotherapy
MIR635	0.00271323	-10.4668	Post- down vs Pre-chemotherapy
FBXO44	0.00272861	-2.92725	Post- down vs Pre-chemotherapy
MACF1	0.00273214	-2.43366	Post- down vs Pre-chemotherapy
KANSL2	0.00274355	-2.76662	Post- down vs Pre-chemotherapy
SAMD9	0.00275141	-3.24356	Post- down vs Pre-chemotherapy
HCP5	0.00276158	-2.9852	Post- down vs Pre-chemotherapy
DCUN1D4	0.00276374	-3.18327	Post- down vs Pre-chemotherapy
DLAT	0.00276923	-2.7397	Post- down vs Pre-chemotherapy
UBR3	0.00278262	-2.48594	Post- down vs Pre-chemotherapy
HAUS5	0.00278721	-3.13586	Post- down vs Pre-chemotherapy
REST	0.00279698	-2.22395	Post- down vs Pre-chemotherapy
PAFAH2	0.00279738	-3.28352	Post- down vs Pre-chemotherapy
MFSD9	0.00285263	-3.0511	Post- down vs Pre-chemotherapy
RUNX1	0.00285585	-2.09941	Post- down vs Pre-chemotherapy
WARS	0.00285958	-3.10252	Post- down vs Pre-chemotherapy
HSPA13	0.00287157	-3.89134	Post- down vs Pre-chemotherapy
CDK19	0.00289736	-2.33824	Post- down vs Pre-chemotherapy
HIST1H4D	0.00290983	-6.91748	Post- down vs Pre-chemotherapy
MIR139	0.00291711	6.78738	Post- up vs Pre-chemotherapy
CLPTM1L	0.00294108	-3.78977	Post- down vs Pre-chemotherapy
ZNF410	0.00295486	-4.39792	Post- down vs Pre-chemotherapy
DYNLL1	0.00295844	-2.34407	Post- down vs Pre-chemotherapy
SCO1	0.00296575	-3.71541	Post- down vs Pre-chemotherapy
NICN1	0.00297267	-4.20553	Post- down vs Pre-chemotherapy
POLR3F	0.00299096	-4.58685	Post- down vs Pre-chemotherapy
SDAD1	0.00299124	-2.63676	Post- down vs Pre-chemotherapy
CTDSPL2	0.00300082	-3.6693	Post- down vs Pre-chemotherapy
NOL8	0.00300673	-2.8942	Post- down vs Pre-chemotherapy
UCKL1	0.00300693	-3.84716	Post- down vs Pre-chemotherapy
MYBL2	0.00303921	-3.38889	Post- down vs Pre-chemotherapy
PGM2L1	0.00306627	-2.18415	Post- down vs Pre-chemotherapy
CWC22	0.00307056	-2.81337	Post- down vs Pre-chemotherapy
TOP1	0.00307621	-3.36032	Post- down vs Pre-chemotherapy
SRI	0.00308329	-3.78688	Post- down vs Pre-chemotherapy
SPG21	0.00308992	-3.13069	Post- down vs Pre-chemotherapy
ZNF641	0.00310693	-3.45256	Post- down vs Pre-chemotherapy
CAP2	0.0031188	-4.80886	Post- down vs Pre-chemotherapy
XPR1	0.00312596	-3.07191	Post- down vs Pre-chemotherapy
ATF7IP2	0.00313242	-3.68819	Post- down vs Pre-chemotherapy
RINT1	0.00316929	-4.66597	Post- down vs Pre-chemotherapy
GALNT3	0.00317319	-2.8689	Post- down vs Pre-chemotherapy
SUGT1	0.00317437	-2.62795	Post- down vs Pre-chemotherapy
APOL6	0.00318689	-2.41228	Post- down vs Pre-chemotherapy
ZSCAN32	0.00320718	-4.13721	Post- down vs Pre-chemotherapy
CXCL10	0.00321808	-5.67342	Post- down vs Pre-chemotherapy
CTSL	0.00323584	-4.43055	Post- down vs Pre-chemotherapy
ZNF816	0.00324668	-5.27339	Post- down vs Pre-chemotherapy
AGGF1	0.00325708	-3.64635	Post- down vs Pre-chemotherapy
CEP85	0.00325803	-2.93019	Post- down vs Pre-chemotherapy
MPP5	0.00325903	-3.04035	Post- down vs Pre-chemotherapy
LAX1	0.00326951	-4.25778	Post- down vs Pre-chemotherapy
OSBP	0.00327236	-3.43288	Post- down vs Pre-chemotherapy
C7orf43	0.00327768	-2.69747	Post- down vs Pre-chemotherapy
ZNF107	0.00328475	-2.94145	Post- down vs Pre-chemotherapy
SS18L1	0.0032849	-2.60929	Post- down vs Pre-chemotherapy
INADL	0.00329186	-2.38339	Post- down vs Pre-chemotherapy
BCL11B	0.00332719	-2.59109	Post- down vs Pre-chemotherapy
SACM1L	0.003336	-3.28153	Post- down vs Pre-chemotherapy
MIR199A1	0.00335195	2.91653	Post- up vs Pre-chemotherapy
CCNYL1	0.00335415	-2.95001	Post- down vs Pre-chemotherapy
MYO1F	0.00338363	-2.12866	Post- down vs Pre-chemotherapy
ZADH2	0.00338536	-2.71804	Post- down vs Pre-chemotherapy
TRMT12	0.00339271	-5.12256	Post- down vs Pre-chemotherapy
DCAF5	0.00340072	-4.46555	Post- down vs Pre-chemotherapy
CEP152	0.00340612	-3.34377	Post- down vs Pre-chemotherapy
ZNF567	0.00343036	-6.07778	Post- down vs Pre-chemotherapy
KAL1	0.00343138	-2.77816	Post- down vs Pre-chemotherapy
NGRN	0.00343979	-4.01695	Post- down vs Pre-chemotherapy
ATP6AP2	0.00344149	-3.34447	Post- down vs Pre-chemotherapy

LATS1	0.00344555	-3.08917	Post- down vs Pre-chemotherapy
ALG12	0.00345199	-3.93816	Post- down vs Pre-chemotherapy
PSMG1	0.00345307	-4.81923	Post- down vs Pre-chemotherapy
GGA3	0.00345918	-2.17858	Post- down vs Pre-chemotherapy
CEP104	0.00347313	-2.38332	Post- down vs Pre-chemotherapy
INSR	0.00347787	-3.0665	Post- down vs Pre-chemotherapy
EXD2	0.00349185	-2.64431	Post- down vs Pre-chemotherapy
GBP5	0.00350418	-4.05054	Post- down vs Pre-chemotherapy
SIPA1L3	0.00354483	-2.06612	Post- down vs Pre-chemotherapy
C1orf131	0.00354943	-4.3866	Post- down vs Pre-chemotherapy
BAHD1	0.00355163	-2.79224	Post- down vs Pre-chemotherapy
RCHY1	0.00355558	-3.15402	Post- down vs Pre-chemotherapy
MIR145	0.00355578	2.93866	Post- up vs Pre-chemotherapy
ERAL1	0.00356287	-3.92392	Post- down vs Pre-chemotherapy
SNORD113-9	0.00356865	2.57661	Post- up vs Pre-chemotherapy
TRIM41	0.0036154	-2.60074	Post- down vs Pre-chemotherapy
NSMCE4A	0.0036179	-2.85526	Post- down vs Pre-chemotherapy
SYTL1	0.00363076	-3.74578	Post- down vs Pre-chemotherapy
FUNDC2	0.0036312	-4.84111	Post- down vs Pre-chemotherapy
POLM	0.00363518	-3.60006	Post- down vs Pre-chemotherapy
LOC150381	0.00365446	-8.4762	Post- down vs Pre-chemotherapy
CIZ1	0.00365822	-2.61931	Post- down vs Pre-chemotherapy
ELK2AP	0.003662	-5.21995	Post- down vs Pre-chemotherapy
FMNL2	0.00367554	-2.53998	Post- down vs Pre-chemotherapy
PXK	0.00370462	-3.65337	Post- down vs Pre-chemotherapy
SPICE1	0.00372419	-4.09287	Post- down vs Pre-chemotherapy
EMC6	0.00374302	-6.22661	Post- down vs Pre-chemotherapy
RNF19A	0.00375244	-2.42546	Post- down vs Pre-chemotherapy
APAF1	0.00377053	-2.83039	Post- down vs Pre-chemotherapy
ST3GAL4	0.00380342	-2.72942	Post- down vs Pre-chemotherapy
CD37	0.00381891	-4.21619	Post- down vs Pre-chemotherapy
MIR99A	0.00382037	2.78211	Post- up vs Pre-chemotherapy
MOB3C	0.00383499	-4.90113	Post- down vs Pre-chemotherapy
RNASEL	0.00387633	-4.19803	Post- down vs Pre-chemotherapy
DGKQ	0.00387883	-2.37612	Post- down vs Pre-chemotherapy
MCM3AP	0.00388434	-2.57663	Post- down vs Pre-chemotherapy
FNDC3B	0.00388728	-2.78836	Post- down vs Pre-chemotherapy
ACACA	0.00388985	-2.14173	Post- down vs Pre-chemotherapy
DNAJC19	0.00390262	-5.39088	Post- down vs Pre-chemotherapy
FAM83H-AS1	0.00393151	-3.3337	Post- down vs Pre-chemotherapy
URGCP	0.00394689	-2.8899	Post- down vs Pre-chemotherapy
PPP1R16B	0.00394789	-2.46308	Post- down vs Pre-chemotherapy
ENO2	0.00396208	-4.7705	Post- down vs Pre-chemotherapy
ZNF518A	0.00400099	-2.3465	Post- down vs Pre-chemotherapy
LOC90784	0.00400392	-2.77663	Post- down vs Pre-chemotherapy
MIR146B	0.00401379	2.82759	Post- up vs Pre-chemotherapy
GABPB1	0.00402526	-5.56426	Post- down vs Pre-chemotherapy
CCDC144A	0.00402586	-3.15223	Post- down vs Pre-chemotherapy
ITIH5	0.00402829	3.00948	Post- up vs Pre-chemotherapy
CISD1	0.00403683	-3.8643	Post- down vs Pre-chemotherapy
NBEA	0.00405418	-3.17705	Post- down vs Pre-chemotherapy
TIRAP	0.00407221	-3.34833	Post- down vs Pre-chemotherapy
FUS	0.00409244	-2.68258	Post- down vs Pre-chemotherapy
MDM1	0.00410919	-2.80672	Post- down vs Pre-chemotherapy
TMEM263	0.00411682	-2.89047	Post- down vs Pre-chemotherapy
ITK	0.00411828	-3.85289	Post- down vs Pre-chemotherapy
CYR61	0.00412066	2.43814	Post- up vs Pre-chemotherapy
SPAG1	0.00412112	-2.50223	Post- down vs Pre-chemotherapy
SNHG1	0.00413061	-5.92104	Post- down vs Pre-chemotherapy
HERC2P3	0.00413252	-6.1722	Post- down vs Pre-chemotherapy
SNORD114-25	0.00413829	2.24045	Post- up vs Pre-chemotherapy
SNAPC3	0.0041396	-2.85479	Post- down vs Pre-chemotherapy
RPAP1	0.00414416	-2.90696	Post- down vs Pre-chemotherapy
COA7	0.00414642	-3.77395	Post- down vs Pre-chemotherapy
CHORDC1	0.00414874	-3.18886	Post- down vs Pre-chemotherapy
RMI2	0.00415396	-5.20572	Post- down vs Pre-chemotherapy
SREBF1	0.00417709	-2.51815	Post- down vs Pre-chemotherapy
AGO2	0.0041808	-2.39504	Post- down vs Pre-chemotherapy
ARL4A	0.00418237	-2.87129	Post- down vs Pre-chemotherapy
ZNF138	0.00418715	-3.21528	Post- down vs Pre-chemotherapy
MX2	0.00418841	-3.42936	Post- down vs Pre-chemotherapy
FAM35A	0.00420078	-3.55818	Post- down vs Pre-chemotherapy
TMEM87B	0.00421571	-2.5135	Post- down vs Pre-chemotherapy

BAZ2B	0.00422306	-2.32373	Post- down vs Pre-chemotherapy
RNGTT	0.00422432	-3.10491	Post- down vs Pre-chemotherapy
BHLHE22	0.00423972	2.24707	Post- up vs Pre-chemotherapy
MIR324	0.00425092	4.79128	Post- up vs Pre-chemotherapy
MCEE	0.00425345	-4.88645	Post- down vs Pre-chemotherapy
POLR2D	0.00426685	-3.53457	Post- down vs Pre-chemotherapy
EIF5	0.00427294	-2.48497	Post- down vs Pre-chemotherapy
CDS2	0.00428091	-2.65161	Post- down vs Pre-chemotherapy
SFMBT2	0.00428423	-2.06531	Post- down vs Pre-chemotherapy
KDM4C	0.00429579	-2.33008	Post- down vs Pre-chemotherapy
ARV1	0.00429974	-4.29711	Post- down vs Pre-chemotherapy
EPB41L5	0.00430056	-2.17803	Post- down vs Pre-chemotherapy
CMPK2	0.00430735	-2.43593	Post- down vs Pre-chemotherapy
ZBTB33	0.00431085	-2.90037	Post- down vs Pre-chemotherapy
NABP2	0.00431197	-4.88513	Post- down vs Pre-chemotherapy
UCHL3	0.00431555	-3.39501	Post- down vs Pre-chemotherapy
AMPD2	0.00432476	-2.84095	Post- down vs Pre-chemotherapy
RBAK	0.00437252	-3.17319	Post- down vs Pre-chemotherapy
UBA5	0.00437707	-3.46056	Post- down vs Pre-chemotherapy
MSH2	0.0043807	-3.98628	Post- down vs Pre-chemotherapy
KLF12	0.00439913	-2.31713	Post- down vs Pre-chemotherapy
COPB2	0.00444478	-3.21376	Post- down vs Pre-chemotherapy
NUB1	0.00445991	-2.90545	Post- down vs Pre-chemotherapy
ZNF224	0.00446758	-3.49134	Post- down vs Pre-chemotherapy
KCTD21	0.00446922	-2.60073	Post- down vs Pre-chemotherapy
STAT3	0.00447857	-2.00205	Post- down vs Pre-chemotherapy
FAM188A	0.00449145	-3.12837	Post- down vs Pre-chemotherapy
MFSD11	0.00449199	-3.36926	Post- down vs Pre-chemotherapy
PLOD2	0.00452299	-2.99339	Post- down vs Pre-chemotherapy
PARD6B	0.00453652	-3.11543	Post- down vs Pre-chemotherapy
ZNF3	0.00454686	-2.35334	Post- down vs Pre-chemotherapy
ATXN7L3	0.00455044	-2.88699	Post- down vs Pre-chemotherapy
GPD1L	0.00455166	-3.46092	Post- down vs Pre-chemotherapy
LCMT1	0.00455651	-4.10486	Post- down vs Pre-chemotherapy
ATP13A3	0.00460169	-3.15109	Post- down vs Pre-chemotherapy
JARID2	0.00462125	-2.91081	Post- down vs Pre-chemotherapy
PAN2	0.00462138	-4.48996	Post- down vs Pre-chemotherapy
PTP4A3	0.00464553	-2.77789	Post- down vs Pre-chemotherapy
SLC9A7	0.00466359	-2.72545	Post- down vs Pre-chemotherapy
FHDC1	0.00466507	-2.55759	Post- down vs Pre-chemotherapy
PPAP2A	0.00469584	-3.96057	Post- down vs Pre-chemotherapy
SNX11	0.00469954	-3.43994	Post- down vs Pre-chemotherapy
KLHL3	0.00470951	-2.43941	Post- down vs Pre-chemotherapy
CDC16	0.00472094	-2.42755	Post- down vs Pre-chemotherapy
EHMT1	0.00472984	-2.67114	Post- down vs Pre-chemotherapy
CD96	0.00473695	-4.21528	Post- down vs Pre-chemotherapy
MIOS	0.00477812	-2.10802	Post- down vs Pre-chemotherapy
HLA-DPB1	0.00477853	-2.10086	Post- down vs Pre-chemotherapy
SLC45A4	0.00477898	-2.34712	Post- down vs Pre-chemotherapy
YEATS2	0.00478464	-3.37593	Post- down vs Pre-chemotherapy
LOC100289230	0.00478947	-2.80403	Post- down vs Pre-chemotherapy
SENP5	0.00478982	-2.86501	Post- down vs Pre-chemotherapy
MVB12B	0.0047905	-2.62686	Post- down vs Pre-chemotherapy
RPSAP58	0.00479214	-4.68667	Post- down vs Pre-chemotherapy
LINC00342	0.00482207	-3.51231	Post- down vs Pre-chemotherapy
DNAJB9	0.00485696	-3.28196	Post- down vs Pre-chemotherapy
MRPS25	0.00485894	-2.79625	Post- down vs Pre-chemotherapy
NLRC5	0.00488995	-3.05598	Post- down vs Pre-chemotherapy
ZMYM2	0.00489161	-2.07717	Post- down vs Pre-chemotherapy
TNRC6A	0.0049062	-2.89936	Post- down vs Pre-chemotherapy
COG5	0.00491061	-2.75393	Post- down vs Pre-chemotherapy
METTL23	0.00494121	-2.88459	Post- down vs Pre-chemotherapy
NAA25	0.00496545	-2.66538	Post- down vs Pre-chemotherapy
MANEA	0.00499165	-3.21509	Post- down vs Pre-chemotherapy
RNF4	0.00499314	-2.65491	Post- down vs Pre-chemotherapy
PLAA	0.00499316	-3.909	Post- down vs Pre-chemotherapy
TRIM14	0.00501455	-2.11174	Post- down vs Pre-chemotherapy
EPHX2	0.00501656	-4.69329	Post- down vs Pre-chemotherapy
ACTR6	0.00502373	-3.50141	Post- down vs Pre-chemotherapy
CDC6	0.00506518	-3.35049	Post- down vs Pre-chemotherapy
IRF4	0.0050864	-3.09411	Post- down vs Pre-chemotherapy
FBXO3	0.00509248	-4.39876	Post- down vs Pre-chemotherapy
TIA1	0.00509572	-3.25165	Post- down vs Pre-chemotherapy

MTFR1L	0.00509942	-3.74569	Post- down vs Pre-chemotherapy
TJP3	0.0051041	-2.14401	Post- down vs Pre-chemotherapy
MIR34C	0.00510643	7.53142	Post- up vs Pre-chemotherapy
PAPSS1	0.00511292	-3.51774	Post- down vs Pre-chemotherapy
MLLT6	0.00511604	-2.08938	Post- down vs Pre-chemotherapy
PSIP1	0.0051189	-2.86529	Post- down vs Pre-chemotherapy
TBC1D8B	0.00512386	-2.50201	Post- down vs Pre-chemotherapy
PPM1K	0.00514237	-2.77622	Post- down vs Pre-chemotherapy
ABCB7	0.00514877	-3.62486	Post- down vs Pre-chemotherapy
CRK	0.00516293	-2.3274	Post- down vs Pre-chemotherapy
SMG9	0.00516444	-3.24735	Post- down vs Pre-chemotherapy
TIMM21	0.00516683	-4.23121	Post- down vs Pre-chemotherapy
CYB561D1	0.00517497	-2.88661	Post- down vs Pre-chemotherapy
DDX60L	0.00518427	-2.38262	Post- down vs Pre-chemotherapy
CD40	0.0051849	-3.26906	Post- down vs Pre-chemotherapy
CCDC127	0.00518827	-2.96341	Post- down vs Pre-chemotherapy
ECT2	0.00519961	-3.3328	Post- down vs Pre-chemotherapy
DNA2	0.00521757	-3.4372	Post- down vs Pre-chemotherapy
TCOF1	0.00524107	-2.61452	Post- down vs Pre-chemotherapy
ZNF808	0.00524927	-3.48109	Post- down vs Pre-chemotherapy
COX7A2	0.00526525	-6.42699	Post- down vs Pre-chemotherapy
MBD5	0.00528048	-3.45919	Post- down vs Pre-chemotherapy
BECN1	0.00529174	-4.04835	Post- down vs Pre-chemotherapy
FAM111A	0.00530464	-2.49873	Post- down vs Pre-chemotherapy
FEZ2	0.00531143	-4.54206	Post- down vs Pre-chemotherapy
DRAM1	0.00532316	-2.14555	Post- down vs Pre-chemotherapy
ZNF721	0.0053294	-2.68045	Post- down vs Pre-chemotherapy
MLXIP	0.00535781	-2.15828	Post- down vs Pre-chemotherapy
ACAD9	0.00535858	-4.06799	Post- down vs Pre-chemotherapy
CMAS	0.00536375	-4.67195	Post- down vs Pre-chemotherapy
PGM3	0.00538857	-2.66141	Post- down vs Pre-chemotherapy
PIR	0.00542	-5.36425	Post- down vs Pre-chemotherapy
HPS5	0.00543569	-2.60822	Post- down vs Pre-chemotherapy
OGT	0.0054479	-2.30216	Post- down vs Pre-chemotherapy
RUNDC1	0.00547186	-3.06437	Post- down vs Pre-chemotherapy
RABGAP1L	0.0054766	-2.3437	Post- down vs Pre-chemotherapy
TLK1	0.00548081	-2.21243	Post- down vs Pre-chemotherapy
TULP3	0.00548819	-3.36619	Post- down vs Pre-chemotherapy
DCTN3	0.00552184	-5.25061	Post- down vs Pre-chemotherapy
ZNF146	0.0055261	-2.28862	Post- down vs Pre-chemotherapy
ZKSCAN3	0.00556583	-3.54409	Post- down vs Pre-chemotherapy
VIPAS39	0.00557259	-3.22273	Post- down vs Pre-chemotherapy
INTS5	0.00557299	-2.17887	Post- down vs Pre-chemotherapy
LINC00997	0.00558142	-2.99808	Post- down vs Pre-chemotherapy
PCBP1	0.00560822	-2.11217	Post- down vs Pre-chemotherapy
LOC100129917	0.00560835	-3.33014	Post- down vs Pre-chemotherapy
PON2	0.00560872	-3.77644	Post- down vs Pre-chemotherapy
STAM	0.00561077	-2.40122	Post- down vs Pre-chemotherapy
KLF13	0.00562089	-2.64513	Post- down vs Pre-chemotherapy
NDUFA12	0.00562862	-9.43631	Post- down vs Pre-chemotherapy
TOP3B	0.00563241	-2.71542	Post- down vs Pre-chemotherapy
ZNF605	0.00564358	-2.90167	Post- down vs Pre-chemotherapy
GEN1	0.00564725	-3.91238	Post- down vs Pre-chemotherapy
ZNF397	0.00567536	-3.57165	Post- down vs Pre-chemotherapy
ARMC8	0.00567608	-2.91372	Post- down vs Pre-chemotherapy
KIAA1109	0.0056912	-2.16091	Post- down vs Pre-chemotherapy
NR4A1	0.0056925	4.24386	Post- up vs Pre-chemotherapy
IL6R	0.00570772	-2.42981	Post- down vs Pre-chemotherapy
NSUN2	0.00572518	-2.68146	Post- down vs Pre-chemotherapy
DHX8	0.00572916	-3.28172	Post- down vs Pre-chemotherapy
NCAPD3	0.00572972	-2.35781	Post- down vs Pre-chemotherapy
IPW	0.00574838	-3.79793	Post- down vs Pre-chemotherapy
DOCK11	0.00575174	-2.64318	Post- down vs Pre-chemotherapy
LAIR1	0.00578073	-3.12571	Post- down vs Pre-chemotherapy
CD2	0.00578953	-3.66335	Post- down vs Pre-chemotherapy
PDK3	0.00579223	-2.44942	Post- down vs Pre-chemotherapy
TMEM57	0.00580378	-3.21732	Post- down vs Pre-chemotherapy
HES1	0.00580475	-3.43883	Post- down vs Pre-chemotherapy
ITGBL1	0.00582451	-2.92326	Post- down vs Pre-chemotherapy
CCDC146	0.00584759	-2.78213	Post- down vs Pre-chemotherapy
CLMN	0.00586659	-2.18237	Post- down vs Pre-chemotherapy
MIR27B	0.00586745	3.96089	Post- up vs Pre-chemotherapy
IGSF3	0.00587873	-2.27266	Post- down vs Pre-chemotherapy

TMEM198B	0.00588283	-3.27683	Post- down vs Pre-chemotherapy
RALGAPA1	0.00591552	-2.71023	Post- down vs Pre-chemotherapy
DENND4B	0.0059245	-2.69338	Post- down vs Pre-chemotherapy
ATG9A	0.0059319	-3.62771	Post- down vs Pre-chemotherapy
CERK	0.00593335	-2.98049	Post- down vs Pre-chemotherapy
EDEM2	0.00594506	-3.55033	Post- down vs Pre-chemotherapy
LTF	0.00596164	-3.82058	Post- down vs Pre-chemotherapy
ARRDC4	0.00597789	-3.48422	Post- down vs Pre-chemotherapy
SELL	0.00599963	-3.99162	Post- down vs Pre-chemotherapy
TMEM68	0.00606474	-2.91728	Post- down vs Pre-chemotherapy
KIAA0040	0.00607467	-3.03104	Post- down vs Pre-chemotherapy
WDR7	0.00608726	-2.23078	Post- down vs Pre-chemotherapy
C21orf91	0.0060918	-2.65607	Post- down vs Pre-chemotherapy
ZC3H14	0.00609901	-2.5108	Post- down vs Pre-chemotherapy
ADCY6	0.00610813	-2.03062	Post- down vs Pre-chemotherapy
LYZ	0.00612719	-4.72032	Post- down vs Pre-chemotherapy
TTC9C	0.00612738	-6.36616	Post- down vs Pre-chemotherapy
TGDS	0.00613042	-4.50676	Post- down vs Pre-chemotherapy
PMS1	0.00613063	-3.20178	Post- down vs Pre-chemotherapy
WHSC1L1	0.00613078	-2.24298	Post- down vs Pre-chemotherapy
MON2	0.00613236	-2.85835	Post- down vs Pre-chemotherapy
ZNF274	0.00613473	-4.37687	Post- down vs Pre-chemotherapy
CD24	0.00614565	-6.21924	Post- down vs Pre-chemotherapy
THOC2	0.00615179	-3.30325	Post- down vs Pre-chemotherapy
PLCB2	0.00619715	-2.72681	Post- down vs Pre-chemotherapy
RAB27A	0.0062004	-2.79884	Post- down vs Pre-chemotherapy
TIGD7	0.00620095	-3.9682	Post- down vs Pre-chemotherapy
CXorf23	0.00621815	-3.08963	Post- down vs Pre-chemotherapy
MCPH1	0.00622173	-2.89266	Post- down vs Pre-chemotherapy
ARFGEF1	0.00625475	-2.15078	Post- down vs Pre-chemotherapy
MYO19	0.00625509	-3.10505	Post- down vs Pre-chemotherapy
GUF1	0.00626419	-4.32282	Post- down vs Pre-chemotherapy
ZDHHC13	0.00628081	-4.18681	Post- down vs Pre-chemotherapy
CPOX	0.0062826	-3.06614	Post- down vs Pre-chemotherapy
FAM214B	0.00629925	-2.19472	Post- down vs Pre-chemotherapy
SPG20	0.00629998	-3.72804	Post- down vs Pre-chemotherapy
NDRG3	0.00631234	-2.37654	Post- down vs Pre-chemotherapy
PPP1R9A	0.00634451	-2.46329	Post- down vs Pre-chemotherapy
SLC30A7	0.00635841	-2.03451	Post- down vs Pre-chemotherapy
AASDH	0.00635983	-3.62111	Post- down vs Pre-chemotherapy
TIGIT	0.00636834	-3.77051	Post- down vs Pre-chemotherapy
SCAF11	0.0063793	-2.03331	Post- down vs Pre-chemotherapy
ITGAL	0.00639665	-3.38541	Post- down vs Pre-chemotherapy
GGT1	0.00640714	-3.24632	Post- down vs Pre-chemotherapy
SYNRG	0.00640731	-2.56481	Post- down vs Pre-chemotherapy
COMMD5	0.00641716	-3.45012	Post- down vs Pre-chemotherapy
DCLRE1C	0.00644553	-3.03667	Post- down vs Pre-chemotherapy
ZNF75D	0.00645877	-3.77172	Post- down vs Pre-chemotherapy
PSEN1	0.00646581	-3.59788	Post- down vs Pre-chemotherapy
EXOC6	0.00648923	-2.55377	Post- down vs Pre-chemotherapy
RUFY2	0.00650283	-2.52298	Post- down vs Pre-chemotherapy
SRP54	0.00652773	-4.30997	Post- down vs Pre-chemotherapy
FER	0.00653094	-5.12983	Post- down vs Pre-chemotherapy
EFTUD1	0.00654364	-3.46969	Post- down vs Pre-chemotherapy
NUP35	0.00654609	-4.00251	Post- down vs Pre-chemotherapy
GPT2	0.00656338	-2.46334	Post- down vs Pre-chemotherapy
VGLL4	0.00657372	-2.14922	Post- down vs Pre-chemotherapy
C3orf38	0.00658332	-4.59581	Post- down vs Pre-chemotherapy
MMADHC	0.00659044	-3.41103	Post- down vs Pre-chemotherapy
AMD1	0.00660467	-3.18821	Post- down vs Pre-chemotherapy
NKRF	0.00660752	-4.12327	Post- down vs Pre-chemotherapy
DLD	0.00660829	-2.99284	Post- down vs Pre-chemotherapy
DPM1	0.00660893	-3.16563	Post- down vs Pre-chemotherapy
MIR452	0.00661447	5.32163	Post- up vs Pre-chemotherapy
TIMM8B	0.00662936	-5.49645	Post- down vs Pre-chemotherapy
RBM12	0.00663214	-2.48888	Post- down vs Pre-chemotherapy
TRPV1	0.00666432	-2.19697	Post- down vs Pre-chemotherapy
C9orf156	0.00667053	-3.14633	Post- down vs Pre-chemotherapy
RAB11A	0.00667994	-2.65297	Post- down vs Pre-chemotherapy
ZFP41	0.00672839	-2.60045	Post- down vs Pre-chemotherapy
LPCAT4	0.0067506	-3.14156	Post- down vs Pre-chemotherapy
ZNF37A	0.00675136	-2.70123	Post- down vs Pre-chemotherapy
AGO4	0.00675803	-2.54464	Post- down vs Pre-chemotherapy

ANLN	0.0067736	-2.48424	Post- down vs Pre-chemotherapy
ANKRA2	0.00677901	-5.13684	Post- down vs Pre-chemotherapy
GTF2F1	0.00680302	-2.67088	Post- down vs Pre-chemotherapy
BPTF	0.00680524	-2.40179	Post- down vs Pre-chemotherapy
ADARB1	0.00683664	-2.40785	Post- down vs Pre-chemotherapy
AK6	0.00684102	-3.66747	Post- down vs Pre-chemotherapy
PSME4	0.00686025	-2.65008	Post- down vs Pre-chemotherapy
TUBA1C	0.00690378	-4.07136	Post- down vs Pre-chemotherapy
LOC727896	0.0069135	-4.26517	Post- down vs Pre-chemotherapy
RSL24D1	0.00692175	-4.22456	Post- down vs Pre-chemotherapy
HOOK1	0.00692912	-2.2948	Post- down vs Pre-chemotherapy
ARL14EP	0.00693186	-2.94164	Post- down vs Pre-chemotherapy
NPIPB5	0.0069592	-3.51171	Post- down vs Pre-chemotherapy
DEPTOR	0.00696453	-3.37804	Post- down vs Pre-chemotherapy
LYSMD2	0.00697316	-3.92367	Post- down vs Pre-chemotherapy
LOC101927151	0.00698254	-2.82695	Post- down vs Pre-chemotherapy
MBLAC2	0.00698313	-2.43256	Post- down vs Pre-chemotherapy
SUPT4H1	0.00698391	-5.29906	Post- down vs Pre-chemotherapy
TSFM	0.00698612	-3.6248	Post- down vs Pre-chemotherapy
NCS1	0.00698942	-2.24058	Post- down vs Pre-chemotherapy
KIAA0753	0.0070055	-2.89434	Post- down vs Pre-chemotherapy
PAPOLG	0.00703785	-2.5907	Post- down vs Pre-chemotherapy
LOC100129361	0.00706946	-2.38405	Post- down vs Pre-chemotherapy
WHSC1	0.00709219	-2.31582	Post- down vs Pre-chemotherapy
ZNF577	0.00712301	-2.61386	Post- down vs Pre-chemotherapy
CENPJ	0.0071292	-4.26789	Post- down vs Pre-chemotherapy
VPS11	0.00713313	-3.79147	Post- down vs Pre-chemotherapy
KAT2B	0.00714052	-3.07236	Post- down vs Pre-chemotherapy
CIITA	0.00715602	-2.80664	Post- down vs Pre-chemotherapy
KIAA0922	0.00715801	-2.75259	Post- down vs Pre-chemotherapy
BNIP3	0.00718484	-3.47193	Post- down vs Pre-chemotherapy
ABT1	0.00718759	-2.23211	Post- down vs Pre-chemotherapy
MIRLET7B	0.00721887	2.30259	Post- up vs Pre-chemotherapy
SMN2	0.00727382	-2.92057	Post- down vs Pre-chemotherapy
WASF2	0.00727591	-2.0457	Post- down vs Pre-chemotherapy
TLR5	0.00728046	-3.09547	Post- down vs Pre-chemotherapy
ARPC5L	0.00728326	-5.10874	Post- down vs Pre-chemotherapy
SPRTN	0.00729247	-2.83672	Post- down vs Pre-chemotherapy
TC2N	0.00729582	-4.28613	Post- down vs Pre-chemotherapy
ZNF83	0.00729821	-3.33807	Post- down vs Pre-chemotherapy
AMBRA1	0.0072995	-2.75693	Post- down vs Pre-chemotherapy
WDR12	0.00731452	-3.76268	Post- down vs Pre-chemotherapy
CDCA8	0.00732128	-3.49525	Post- down vs Pre-chemotherapy
TMEM170B	0.00732802	-2.78005	Post- down vs Pre-chemotherapy
PLXNC1	0.00734352	-2.56718	Post- down vs Pre-chemotherapy
JUN	0.00734544	2.34712	Post- up vs Pre-chemotherapy
ADAMTS12	0.00734935	-2.74093	Post- down vs Pre-chemotherapy
GBA2	0.00736096	-3.20484	Post- down vs Pre-chemotherapy
ELAVL1	0.00738256	-2.21341	Post- down vs Pre-chemotherapy
PDCD7	0.00740461	-2.43725	Post- down vs Pre-chemotherapy
SKIV2L2	0.00740839	-2.3798	Post- down vs Pre-chemotherapy
UBQLN2	0.0074375	-2.28744	Post- down vs Pre-chemotherapy
DNAJC15	0.00745129	-4.14231	Post- down vs Pre-chemotherapy
APOOL	0.00746405	-2.45765	Post- down vs Pre-chemotherapy
PTPRF	0.007468	-2.4279	Post- down vs Pre-chemotherapy
ARHGAP8	0.00746849	-4.10671	Post- down vs Pre-chemotherapy
PTK2B	0.00747223	-2.28948	Post- down vs Pre-chemotherapy
NAA35	0.00747263	-3.35834	Post- down vs Pre-chemotherapy
SHANK2	0.00748243	-2.5431	Post- down vs Pre-chemotherapy
AKAP9	0.00748579	-2.08922	Post- down vs Pre-chemotherapy
NUP160	0.00749301	-2.20996	Post- down vs Pre-chemotherapy
WDR13	0.00749709	-2.28544	Post- down vs Pre-chemotherapy
CCDC149	0.00749868	-2.44452	Post- down vs Pre-chemotherapy
MYO10	0.00750973	-2.20629	Post- down vs Pre-chemotherapy
UBE2I	0.00751873	-2.92791	Post- down vs Pre-chemotherapy
TICRR	0.00752126	-2.31315	Post- down vs Pre-chemotherapy
ZNF862	0.00752179	-3.13339	Post- down vs Pre-chemotherapy
EIF3J	0.00754161	-3.39594	Post- down vs Pre-chemotherapy
DMXL2	0.00755952	-2.1197	Post- down vs Pre-chemotherapy
RALGPS2	0.00759298	-2.75663	Post- down vs Pre-chemotherapy
ZMAT1	0.00761692	-2.77852	Post- down vs Pre-chemotherapy
RBBP7	0.00765277	-2.59358	Post- down vs Pre-chemotherapy
PARG	0.00766139	-3.00971	Post- down vs Pre-chemotherapy

CTGF	0.00767378	2.13059	Post- up vs Pre-chemotherapy
MAFG	0.00773476	-2.71061	Post- down vs Pre-chemotherapy
KCNN4	0.00781224	-3.08749	Post- down vs Pre-chemotherapy
ZNF91	0.007816	-3.3271	Post- down vs Pre-chemotherapy
CD80	0.00783263	-4.80424	Post- down vs Pre-chemotherapy
LOC374443	0.00786655	-3.88716	Post- down vs Pre-chemotherapy
ZSWIM7	0.00787249	-3.96068	Post- down vs Pre-chemotherapy
RANBP9	0.00787969	-3.18943	Post- down vs Pre-chemotherapy
UBP1	0.00787998	-2.63206	Post- down vs Pre-chemotherapy
COG4	0.00788975	-2.47453	Post- down vs Pre-chemotherapy
TMPPE	0.0079079	-3.30299	Post- down vs Pre-chemotherapy
CASKIN2	0.00792491	-2.05833	Post- down vs Pre-chemotherapy
PARP6	0.00794404	-4.23623	Post- down vs Pre-chemotherapy
ACAD8	0.00794473	-2.18671	Post- down vs Pre-chemotherapy
TOPORS	0.00794613	-2.82753	Post- down vs Pre-chemotherapy
SASH1	0.00794743	-2.02548	Post- down vs Pre-chemotherapy
DNAJC10	0.00797273	-2.58084	Post- down vs Pre-chemotherapy
USP13	0.00798521	-2.66988	Post- down vs Pre-chemotherapy
GUSBP9	0.00800222	-8.74087	Post- down vs Pre-chemotherapy
MED28	0.00800865	-5.82058	Post- down vs Pre-chemotherapy
TPM3P9	0.00800969	-3.9416	Post- down vs Pre-chemotherapy
ITGA1	0.00802258	-2.27104	Post- down vs Pre-chemotherapy
PDCD4	0.00802649	-2.51101	Post- down vs Pre-chemotherapy
ARL16	0.00806455	-4.46383	Post- down vs Pre-chemotherapy
NBEAL1	0.00808687	-2.6799	Post- down vs Pre-chemotherapy
MLKL	0.00808801	-3.96769	Post- down vs Pre-chemotherapy
DIDO1	0.00808845	-2.19803	Post- down vs Pre-chemotherapy
VIMP	0.00810123	-2.63032	Post- down vs Pre-chemotherapy
SORL1	0.00813392	-2.49258	Post- down vs Pre-chemotherapy
RPS18P9	0.00814482	-4.2982	Post- down vs Pre-chemotherapy
ELMO2	0.00814959	-2.94143	Post- down vs Pre-chemotherapy
HADH	0.00815808	-4.07517	Post- down vs Pre-chemotherapy
EHF	0.00816167	-2.85243	Post- down vs Pre-chemotherapy
BBS2	0.00818474	-3.69205	Post- down vs Pre-chemotherapy
XPO1	0.00818558	-3.00517	Post- down vs Pre-chemotherapy
TMEM41A	0.00826302	-3.5139	Post- down vs Pre-chemotherapy
CNOT6	0.00827895	-2.37344	Post- down vs Pre-chemotherapy
NR1D2	0.0082946	-2.33519	Post- down vs Pre-chemotherapy
MEAF6	0.0083024	-2.50004	Post- down vs Pre-chemotherapy
SMC5	0.0083181	-3.25579	Post- down vs Pre-chemotherapy
COIL	0.00832542	-2.64056	Post- down vs Pre-chemotherapy
NLRC3	0.00832705	-2.94477	Post- down vs Pre-chemotherapy
NVL	0.00832769	-2.56	Post- down vs Pre-chemotherapy
ATP11A	0.00833955	-2.13786	Post- down vs Pre-chemotherapy
ZNF655	0.0083501	-2.24013	Post- down vs Pre-chemotherapy
TXNRD1	0.00836213	-3.07738	Post- down vs Pre-chemotherapy
PHOSPHO2	0.0083908	-4.37273	Post- down vs Pre-chemotherapy
MAP7	0.0083962	-2.23456	Post- down vs Pre-chemotherapy
ADM2	0.00841835	-2.44001	Post- down vs Pre-chemotherapy
LINC01004	0.0084193	-2.4234	Post- down vs Pre-chemotherapy
LOC283070	0.00842687	-4.86732	Post- down vs Pre-chemotherapy
KLHL8	0.00843409	-2.77821	Post- down vs Pre-chemotherapy
TOR1AIP1	0.00844272	-2.44327	Post- down vs Pre-chemotherapy
MIR483	0.0084622	4.53192	Post- up vs Pre-chemotherapy
ING1	0.00847233	-2.55092	Post- down vs Pre-chemotherapy
NEMF	0.00847928	-2.75518	Post- down vs Pre-chemotherapy
SETDB1	0.00848846	-2.92072	Post- down vs Pre-chemotherapy
EDRF1	0.00848932	-3.53536	Post- down vs Pre-chemotherapy
UACA	0.00850145	-2.47645	Post- down vs Pre-chemotherapy
C8orf59	0.00850517	-4.83754	Post- down vs Pre-chemotherapy
PHC3	0.00851114	-2.03661	Post- down vs Pre-chemotherapy
NAE1	0.0085123	-4.2818	Post- down vs Pre-chemotherapy
SMARCA4	0.00857486	-2.042	Post- down vs Pre-chemotherapy
ORMDL2	0.00857545	-5.43262	Post- down vs Pre-chemotherapy
ZNF420	0.00859699	-4.18369	Post- down vs Pre-chemotherapy
CDK3	0.00860081	-4.14117	Post- down vs Pre-chemotherapy
ARPC3	0.00860697	-3.18425	Post- down vs Pre-chemotherapy
ZNF845	0.0086131	-3.81267	Post- down vs Pre-chemotherapy
ZNF529	0.00861583	-3.15419	Post- down vs Pre-chemotherapy
SLAMF8	0.0086609	-3.58414	Post- down vs Pre-chemotherapy
GCA	0.00868461	-2.73222	Post- down vs Pre-chemotherapy
TMEM126B	0.00869993	-7.12173	Post- down vs Pre-chemotherapy
CCDC88B	0.00871302	-2.42731	Post- down vs Pre-chemotherapy

MIR663B	0.00873343	2.29806	Post- up vs Pre-chemotherapy
USP28	0.00876503	-2.64364	Post- down vs Pre-chemotherapy
NR0B1	0.0087872	2.67108	Post- up vs Pre-chemotherapy
BHLHE41	0.00881821	-2.43567	Post- down vs Pre-chemotherapy
PHF5A	0.00882825	-4.81192	Post- down vs Pre-chemotherapy
PRKCI	0.00884479	-2.96511	Post- down vs Pre-chemotherapy
KANSL1L	0.00887534	-2.77255	Post- down vs Pre-chemotherapy
CD226	0.00890688	-3.59963	Post- down vs Pre-chemotherapy
GPR65	0.00892464	-3.35087	Post- down vs Pre-chemotherapy
HSPBAP1	0.00893218	-3.55375	Post- down vs Pre-chemotherapy
SCO2	0.0089374	-3.50276	Post- down vs Pre-chemotherapy
PIBF1	0.00894838	-3.93322	Post- down vs Pre-chemotherapy
