# Supplementary material for: Profiling molecular regulators of recurrence in chemorefractory triple-negative breast cancers
Source: Breast Cancer Res. 2019 Aug 5;21:87. doi: 10.1186/s13058-019-1171-7 (PMC6683504; doi:10.1186/s13058-019-1171-7)
Supplement: Supplementary file 6 — Table S5. Pre- and post-NAC upstream regulators (PDF 199 kb) [file 13058_2019_1171_MOESM6_ESM.pdf]

Upstream Regulator	Predicted Activation State	Activation z-score	p-value of overlap
IFNL1	Inhibited	-3.44	0.0000285
STAT3	Activated	2.058	0.0000894
REL	Inhibited	-3.178	0.000161
IFNK	Inhibited	-2	0.000203
IFNE	Inhibited	-2.219	0.000428
PRL	Inhibited	-4	0.00118
STAT1	Inhibited	-3.804	0.00163
MAP4K4	Activated	3.45	0.00173
IRF7	Inhibited	-3.765	0.00193
CD40LG	Inhibited	-2.068	0.00243
XBP1	Inhibited	-3.524	0.00289
IL21	Inhibited	-3.342	0.00324
IFNA1/IFNA13	Inhibited	-2.564	0.00494
BAK1	Inhibited	-2.219	0.00497
CD2	Inhibited	-2.433	0.0056
BCL3	Activated	2.429	0.0056
CD24	Inhibited	-2.152	0.00768
IRF3	Inhibited	-3.048	0.00843
IFNA2	Inhibited	-3.749	0.00982
NKX2-3	Activated	2	0.0104
BAX	Inhibited	-2.433	0.0157
MAPK1	Activated	2.503	0.0163
NFKB1	Inhibited	-2.146	0.0174
IRF5	Inhibited	-2.579	0.0182
FAS	Inhibited	-2.313	0.0205
IRF1	Inhibited	-2.921	0.0239
IRF4	Activated	2.375	0.0277
CSF2	Inhibited	-3.195	0.0315
EBI3	Inhibited	-2.19	0.0361
EBF1	Inhibited	-2.985	0.0366
IL10RA	Activated	3.298	0.0372
ATF4	Inhibited	-2.945	0.0387
NUPR1	Activated	2.685	0.0422
NFE2L2	Inhibited	-3.053	0.0439
MITF	Inhibited	-3.046	0.0472
RELB	Inhibited	-2.219	0.0472
TLR7	Inhibited	-3.067	0.049
