# Supplementary material for: Profiling molecular regulators of recurrence in chemorefractory triple-negative breast cancers
Source: Breast Cancer Res. 2019 Aug 5;21:87. doi: 10.1186/s13058-019-1171-7 (PMC6683504; doi:10.1186/s13058-019-1171-7)
Supplement: Supplementary file 7 — Table S6. 508 genes with differential expression with ≤ 10%FDR between TP53 compound mutants and remaining tumors (n = 52). (PDF 202 kb) [file 13058_2019_1171_MOESM7_ESM.pdf]

Gene Syml	p-value(Compound vs. All Others)	Fold-Change(Compound vs. All Others)	Fold-Change(Compound vs. All Others) (Description)
ELMOD2	5.61E-06	9.73089	Compound up vs All Others
ATF7	1.30E-05	6.32578	Compound up vs All Others
POGK	1.68E-05	8.34768	Compound up vs All Others
POLH	2.64E-05	5.2968	Compound up vs All Others
PRDM4	3.32E-05	8.43474	Compound up vs All Others
NCOA1	4.34E-05	7.57933	Compound up vs All Others
GAR1	4.50E-05	13.4819	Compound up vs All Others
CCZ1	6.33E-05	14.6493	Compound up vs All Others
HMGXB4	7.55E-05	5.68316	Compound up vs All Others
RPRD1A	7.74E-05	7.04897	Compound up vs All Others
RASSF8	7.98E-05	7.57413	Compound up vs All Others
NEK7	8.31E-05	5.99882	Compound up vs All Others
OTUD3	0.000104445	5.37151	Compound up vs All Others
ALG10B	0.00010772	5.86039	Compound up vs All Others
MIER1	0.000113095	5.39711	Compound up vs All Others
MRPL42	0.000127326	6.56628	Compound up vs All Others
ATAD5	0.000155515	7.33371	Compound up vs All Others
TARS2	0.000174444	6.24453	Compound up vs All Others
LATS1	0.000175208	5.09985	Compound up vs All Others
ZMYM2	0.000175602	4.13899	Compound up vs All Others
PNRC2	0.00017839	8.54626	Compound up vs All Others
NACA	0.000180624	6.16847	Compound up vs All Others
GOPC	0.000183983	5.18851	Compound up vs All Others
HM13	0.000184771	6.29079	Compound up vs All Others
VTA1	0.000201665	7.53304	Compound up vs All Others
USB1	0.000227419	5.78361	Compound up vs All Others
STAG1	0.000239173	4.54425	Compound up vs All Others
SEC24A	0.000245943	4.75332	Compound up vs All Others
ATP6V1C1	0.000254842	5.25106	Compound up vs All Others
ANP32A	0.000263276	8.17429	Compound up vs All Others
NELFCD	0.000267438	4.73597	Compound up vs All Others
CCDC14	0.000274305	8.85644	Compound up vs All Others
ARHGAP11	0.000275096	6.42646	Compound up vs All Others
POLR1D	0.000285533	3.90809	Compound up vs All Others
PPP2R3A	0.000288651	5.39146	Compound up vs All Others
EIF3E	0.000296649	10.4838	Compound up vs All Others
ZBTB6	0.000299553	6.99444	Compound up vs All Others
FECH	0.000325714	4.79442	Compound up vs All Others
PROSC	0.000328531	6.55193	Compound up vs All Others
ERF	0.000348699	6.04248	Compound up vs All Others
LRRFIP2	0.000379481	4.83782	Compound up vs All Others
IPO7	0.000383768	4.89402	Compound up vs All Others
PEX1	0.00038661	5.55039	Compound up vs All Others
TMEM87A	0.000389794	5.83868	Compound up vs All Others
NBN	0.000391192	5.92804	Compound up vs All Others
EPM2AIP1	0.000391879	4.38496	Compound up vs All Others
DNM1L	0.00040441	5.76833	Compound up vs All Others
VDAC1	0.00041355	7.76469	Compound up vs All Others
ZMYM1	0.000428819	7.27972	Compound up vs All Others
HINT1	0.00043188	9.64533	Compound up vs All Others
SKIV2L2	0.000440532	6.52762	Compound up vs All Others
RABGGTB	0.000449976	9.77122	Compound up vs All Others
SETDB1	0.000471135	6.2882	Compound up vs All Others
SEMA4C	0.000472307	4.15117	Compound up vs All Others
RABAC1	0.000473669	10.2491	Compound up vs All Others
SLC39A1	0.000474313	6.10939	Compound up vs All Others
RAD1	0.00047922	6.68826	Compound up vs All Others
XPNPEP3	0.000480109	4.03651	Compound up vs All Others
RFWD3	0.000484709	4.8036	Compound up vs All Others
SLC25A16	0.000508741	7.46348	Compound up vs All Others
CCDC50	0.000515139	4.89979	Compound up vs All Others
CCNC	0.000515309	7.07371	Compound up vs All Others
CGGBP1	0.000521433	4.69293	Compound up vs All Others

AP3M1	0.000521991	6.49995	Compound up vs All Others
TSPYL4	0.000522761	5.5169	Compound up vs All Others
BBX	0.000536982	3.69439	Compound up vs All Others
WWTR1	0.000537965	5.4802	Compound up vs All Others
PTP4A1	0.000546047	7.79985	Compound up vs All Others
ODF2	0.00054898	4.45842	Compound up vs All Others
IL13RA1	0.000575174	5.57136	Compound up vs All Others
BET1	0.000592611	7.85445	Compound up vs All Others
GNPNAT1	0.000599632	6.19022	Compound up vs All Others
CDK19	0.000626214	6.25423	Compound up vs All Others
SMAD1	0.000630812	5.12537	Compound up vs All Others
ZNF687	0.000632708	3.62426	Compound up vs All Others
RBM26	0.000640156	5.39315	Compound up vs All Others
CCDC25	0.000653367	5.53087	Compound up vs All Others
TPP2	0.000662637	5.51042	Compound up vs All Others
ATAD2	0.000682734	4.40367	Compound up vs All Others
HOOK3	0.000684401	3.05251	Compound up vs All Others
GOLGA2	0.000692105	5.20144	Compound up vs All Others
MKLN1	0.000696732	3.75879	Compound up vs All Others
SMC3	0.000704347	5.50059	Compound up vs All Others
MTHFD2	0.000705486	7.8832	Compound up vs All Others
NCAPG2	0.000712587	4.7865	Compound up vs All Others
ZNF410	0.000717415	6.49463	Compound up vs All Others
ARPC1A	0.000756057	7.45075	Compound up vs All Others
ISOC2	0.000760162	7.59332	Compound up vs All Others
NUFIP2	0.000779738	3.69062	Compound up vs All Others
SMAD2	0.000783933	3.18567	Compound up vs All Others
EPT1	0.00079316	5.16561	Compound up vs All Others
TBCD	0.000817684	3.49039	Compound up vs All Others
SASS6	0.000825607	7.64845	Compound up vs All Others
AHCTF1	0.000855102	6.93846	Compound up vs All Others
SMARCC1	0.000858203	4.265	Compound up vs All Others
MRPL50	0.000865527	10.9531	Compound up vs All Others
ZNF445	0.000888893	4.53204	Compound up vs All Others
EP400	0.000894095	2.43548	Compound up vs All Others
DUSP16	0.000902195	3.67927	Compound up vs All Others
PHAX	0.000903679	4.74908	Compound up vs All Others
YY1AP1	0.000909479	3.72484	Compound up vs All Others
NDUFS1	0.000909506	6.17927	Compound up vs All Others
HNRNPDL	0.000927882	5.29659	Compound up vs All Others
SPTLC2	0.000930248	3.11471	Compound up vs All Others
HMCES	0.000930446	6.97426	Compound up vs All Others
BAG6	0.000932847	3.18214	Compound up vs All Others
HIST1H2AE	0.000953812	9.32745	Compound up vs All Others
HP1BP3	0.000954008	4.96607	Compound up vs All Others
TMEM209	0.000980835	7.12726	Compound up vs All Others
NCKAP1	0.000982416	4.12042	Compound up vs All Others
TLK1	0.00100529	2.97991	Compound up vs All Others
PTER	0.0010067	7.35721	Compound up vs All Others
SSX2IP	0.0010087	4.27831	Compound up vs All Others
PTP4A3	0.00101237	4.07501	Compound up vs All Others
MEX3A	0.00101397	5.93462	Compound up vs All Others
NSD1	0.00102606	3.63884	Compound up vs All Others
RBFOX2	0.00102912	3.53616	Compound up vs All Others
TRIM37	0.00103912	5.53068	Compound up vs All Others
RNGTT	0.00104545	5.25519	Compound up vs All Others
IDH1	0.00105056	5.79167	Compound up vs All Others
BARD1	0.00105075	4.7519	Compound up vs All Others
DAP3	0.00105807	8.04957	Compound up vs All Others
CDC37	0.0010642	5.89594	Compound up vs All Others
CYP51A1	0.00107364	6.42826	Compound up vs All Others
ZNF33A	0.00107907	5.10654	Compound up vs All Others
KTN1	0.00109043	4.90804	Compound up vs All Others
LSM14A	0.00109469	4.43203	Compound up vs All Others
APEH	0.00109983	4.95316	Compound up vs All Others

STAG2	0.00110755	4.44988	Compound up vs All Others
GOSR2	0.00113137	4.62182	Compound up vs All Others
TRAPPC8	0.0011372	4.50658	Compound up vs All Others
CPQ	0.00114051	9.28379	Compound up vs All Others
RNF130	0.00115116	3.51966	Compound up vs All Others
C17orf80	0.00116545	5.36627	Compound up vs All Others
REST	0.00119994	3.55968	Compound up vs All Others
RFC1	0.00121483	4.66273	Compound up vs All Others
MOB3B	0.00123148	4.76097	Compound up vs All Others
LAPTM4A	0.00124361	5.24872	Compound up vs All Others
BTF3	0.0012472	5.15386	Compound up vs All Others
DRAP1	0.00125329	7.38377	Compound up vs All Others
C18orf25	0.00125387	5.34456	Compound up vs All Others
SNORA38	0.00126274	8.96095	Compound up vs All Others
GABPB1-AS	0.00126781	5.14437	Compound up vs All Others
TNFRSF21	0.0012703	4.35737	Compound up vs All Others
CDC42EP3	0.00127093	4.1761	Compound up vs All Others
GNG10	0.00128616	10.6448	Compound up vs All Others
CENPF	0.0013012	5.2386	Compound up vs All Others
FOXRED2	0.00130637	4.5625	Compound up vs All Others
PSMD14	0.00131235	6.51302	Compound up vs All Others
GALNT3	0.0013157	7.3197	Compound up vs All Others
GRAMD1A	0.00132336	4.30836	Compound up vs All Others
RALBP1	0.00132465	4.81198	Compound up vs All Others
RBM25	0.00132737	5.79848	Compound up vs All Others
FANCA	0.00132747	4.29324	Compound up vs All Others
VPS4B	0.00133525	4.97884	Compound up vs All Others
TRIM44	0.00134158	3.41695	Compound up vs All Others
FAM175B	0.00137792	4.80371	Compound up vs All Others
API5	0.00141174	5.69427	Compound up vs All Others
MIER3	0.00143239	5.58513	Compound up vs All Others
TAX1BP3	0.00143316	6.47034	Compound up vs All Others
C9orf78	0.00143661	6.53496	Compound up vs All Others
PUM2	0.00143958	3.55525	Compound up vs All Others
PPFIBP1	0.00145723	4.01807	Compound up vs All Others
SRP14	0.00147039	6.16252	Compound up vs All Others
ITGA2	0.00148099	4.33525	Compound up vs All Others
G2E3	0.0014837	4.85341	Compound up vs All Others
RPRD1B	0.00148667	4.53159	Compound up vs All Others
MEST	0.00150203	8.26853	Compound up vs All Others
RPL12	0.00150713	6.28057	Compound up vs All Others
MED28	0.00152049	7.30979	Compound up vs All Others
NDUFC2	0.00154035	8.48651	Compound up vs All Others
ROCK2	0.0015426	3.59927	Compound up vs All Others
TRIP6	0.0015485	5.76345	Compound up vs All Others
NPAT	0.00155644	4.27475	Compound up vs All Others
PNO1	0.00157174	6.73325	Compound up vs All Others
MDM2	0.00157368	4.06612	Compound up vs All Others
POLD3	0.00157463	5.29346	Compound up vs All Others
NCBP2	0.00157509	6.44373	Compound up vs All Others
BET1L	0.00159943	4.42215	Compound up vs All Others
SIN3A	0.00160075	3.92741	Compound up vs All Others
RAD21	0.0016176	3.71099	Compound up vs All Others
HIPK3	0.00164926	3.74641	Compound up vs All Others
SRSF2	0.00166773	4.18546	Compound up vs All Others
ZNF337	0.00168463	4.30986	Compound up vs All Others
MAK16	0.00168856	5.09259	Compound up vs All Others
GTF2H1	0.00170083	3.89956	Compound up vs All Others
NBAS	0.0017212	4.05253	Compound up vs All Others
SNX7	0.00172528	6.93433	Compound up vs All Others
CELF1	0.00173064	3.56015	Compound up vs All Others
PAQR3	0.00176149	5.33683	Compound up vs All Others
SPP1	0.00177935	9.23191	Compound up vs All Others
ZNF587B	0.00178425	4.75206	Compound up vs All Others
STARD3	0.00180273	2.94528	Compound up vs All Others

DMTF1	0.00180905	5.54045	Compound up vs All Others
PHF6	0.0018123	3.24266	Compound up vs All Others
RNF7	0.00182069	6.53921	Compound up vs All Others
NMT1	0.0018285	3.25076	Compound up vs All Others
RNF38	0.00182898	3.19151	Compound up vs All Others
ERBB2IP	0.00184058	4.24546	Compound up vs All Others
GFM1	0.00186634	6.13975	Compound up vs All Others
SRP72	0.00187404	4.15466	Compound up vs All Others
RHOC	0.00187405	4.74172	Compound up vs All Others
CAST	0.00187461	4.08876	Compound up vs All Others
MAP1LC3B	0.00192978	6.13961	Compound up vs All Others
RAD18	0.00193636	4.98234	Compound up vs All Others
FCHSD2	0.00193769	4.59579	Compound up vs All Others
BCAT1	0.00194786	4.63897	Compound up vs All Others
FAM135A	0.00195858	4.02439	Compound up vs All Others
CNIH1	0.00197677	6.09017	Compound up vs All Others
RTFDC1	0.00197787	6.85885	Compound up vs All Others
ZCCHC11	0.00198364	4.40636	Compound up vs All Others
ZC3H7A	0.00198862	5.66031	Compound up vs All Others
ICMT	0.00200284	2.75215	Compound up vs All Others
SOAT1	0.00200424	4.42934	Compound up vs All Others
RUFY3	0.00201073	3.84417	Compound up vs All Others
CCPG1	0.00202803	3.72754	Compound up vs All Others
EIF4A3	0.00203329	6.48858	Compound up vs All Others
ACSL4	0.0020609	4.40711	Compound up vs All Others
STMN1	0.00207671	5.58738	Compound up vs All Others
ARHGEF18	0.00208316	2.56876	Compound up vs All Others
SRC	0.0021435	4.25792	Compound up vs All Others
ATP2C1	0.00215161	4.20267	Compound up vs All Others
EP300	0.0021643	3.2069	Compound up vs All Others
ZBTB43	0.00219404	5.30979	Compound up vs All Others
KIF2C	0.00220501	4.60758	Compound up vs All Others
EIF4A1	0.00220886	5.70072	Compound up vs All Others
SRSF10	0.00221595	3.51488	Compound up vs All Others
PEX5	0.00222715	4.41705	Compound up vs All Others
DDIT4	0.00223441	7.22078	Compound up vs All Others
ANAPC7	0.00223874	3.85677	Compound up vs All Others
FZD3	0.00224297	3.77454	Compound up vs All Others
5-Mar	0.00227199	2.95121	Compound up vs All Others
GOLIM4	0.00227542	4.52885	Compound up vs All Others
SLC4A2	0.00228668	2.92665	Compound up vs All Others
MCCC1	0.00228822	4.35932	Compound up vs All Others
SPTLC1	0.00230671	4.74165	Compound up vs All Others
GOLGA3	0.00234801	2.83826	Compound up vs All Others
DMPK	0.00234918	4.02238	Compound up vs All Others
CEP78	0.00234941	5.6664	Compound up vs All Others
PPIC	0.00236723	6.0248	Compound up vs All Others
ATG12	0.0023721	4.02199	Compound up vs All Others
MMGT1	0.00243086	4.88204	Compound up vs All Others
YTHDF1	0.00243422	3.46017	Compound up vs All Others
ZNF146	0.00244168	5.55174	Compound up vs All Others
AP3S2	0.00244716	4.37595	Compound up vs All Others
CSNK1G1	0.00245243	3.48682	Compound up vs All Others
CDC42EP1	0.00245267	5.49678	Compound up vs All Others
SEC22C	0.00245617	4.41364	Compound up vs All Others
ZDHHC21	0.00246579	3.95799	Compound up vs All Others
ATF6	0.00247222	2.87675	Compound up vs All Others
USP46	0.0025058	3.21995	Compound up vs All Others
RPS12	0.00252498	6.2	Compound up vs All Others
EGLN1	0.00254844	2.23977	Compound up vs All Others
TXN2	0.00255269	5.96932	Compound up vs All Others
HIST1H2BC	0.00256755	8.80332	Compound up vs All Others
LAMB1	0.00257796	3.50576	Compound up vs All Others
ARHGAP35	0.00258371	3.46491	Compound up vs All Others
KHDRBS1	0.00259072	3.6588	Compound up vs All Others

TIMM44	0.00260881	4.77364	Compound up vs All Others
CTTNBP2N	0.00260921	3.88316	Compound up vs All Others
TIMP1	0.00260922	5.95595	Compound up vs All Others
PSMD11	0.00260932	4.6925	Compound up vs All Others
CCNA2	0.00261379	5.90959	Compound up vs All Others
P4HA1	0.00261396	4.4545	Compound up vs All Others
CIPC	0.00261702	4.61881	Compound up vs All Others
TIA1	0.00262075	4.39952	Compound up vs All Others
CCT2	0.00262855	5.52692	Compound up vs All Others
E2F1	0.00265005	4.12932	Compound up vs All Others
BAZ1B	0.00267741	3.08287	Compound up vs All Others
AREL1	0.0026798	4.28727	Compound up vs All Others
DPH2	0.00268548	5.58	Compound up vs All Others
CERS6	0.00269788	2.66937	Compound up vs All Others
HDAC2	0.00272196	4.231	Compound up vs All Others
TMEM263	0.00274612	4.75199	Compound up vs All Others
TWSG1	0.00276333	6.23292	Compound up vs All Others
AAMP	0.00277526	4.50935	Compound up vs All Others
NUP43	0.00277755	3.93477	Compound up vs All Others
SKP2	0.00278302	6.17837	Compound up vs All Others
SMAD5	0.00280363	3.69101	Compound up vs All Others
ZBTB8A	0.0028064	2.4412	Compound up vs All Others
YEATS2	0.00280998	3.74384	Compound up vs All Others
AKAP8L	0.00282475	4.09899	Compound up vs All Others
NUP205	0.0028505	3.64367	Compound up vs All Others
YIPF2	0.00285795	5.20777	Compound up vs All Others
MLF2	0.00287145	4.84451	Compound up vs All Others
RNF169	0.00287581	3.28308	Compound up vs All Others
TEX10	0.00288237	4.55985	Compound up vs All Others
DHX40	0.00290372	4.6553	Compound up vs All Others
PTPN12	0.00290443	5.01441	Compound up vs All Others
TPM1	0.00291104	3.65461	Compound up vs All Others
TCF20	0.00291425	3.43323	Compound up vs All Others
ZNF783	0.00292475	3.98066	Compound up vs All Others
LDHB	0.00293429	8.12029	Compound up vs All Others
USP14	0.00294852	5.7792	Compound up vs All Others
CYBB	0.00295859	5.68028	Compound up vs All Others
HIST1H2BD	0.00296316	7.57321	Compound up vs All Others
CD302	0.00297011	5.97192	Compound up vs All Others
NBR1	0.00298186	3.12647	Compound up vs All Others
MBTD1	0.00301191	4.88584	Compound up vs All Others
TXLNA	0.00301721	3.42131	Compound up vs All Others
SNRPD1	0.00302309	8.14791	Compound up vs All Others
LOC64676[illegible]	0.00302429	3.22927	Compound up vs All Others
CCNG2	0.00302945	3.94855	Compound up vs All Others
WRNIP1	0.00303875	4.07989	Compound up vs All Others
CDK4	0.00304831	5.56583	Compound up vs All Others
MIR301A	0.00308006	14.5554	Compound up vs All Others
ZNF609	0.00309659	2.56527	Compound up vs All Others
PLK2	0.00311528	5.76741	Compound up vs All Others
SSFA2	0.00313895	3.19874	Compound up vs All Others
RPS10-NU[illegible]	0.00314004	7.55736	Compound up vs All Others
CAND1	0.00316431	3.12288	Compound up vs All Others
KIAA1143	0.00316488	4.34838	Compound up vs All Others
B4GALT1	0.00316943	3.72533	Compound up vs All Others
MBD2	0.00318315	3.61972	Compound up vs All Others
DPY19L1	0.00318836	3.80874	Compound up vs All Others
SESTD1	0.00321968	3.65826	Compound up vs All Others
MICU2	0.00321972	5.77359	Compound up vs All Others
GNG12	0.00323434	4.45398	Compound up vs All Others
PCGF5	0.00325314	3.83876	Compound up vs All Others
SLC25A40	0.00325367	6.21227	Compound up vs All Others
CIRBP	0.00328432	3.12708	Compound up vs All Others
NARS2	0.00329511	4.14781	Compound up vs All Others
ZBTB47	0.00329962	3.40809	Compound up vs All Others

CASD1	0.00331561	5.60246	Compound up vs All Others
MPDZ	0.00332769	3.74715	Compound up vs All Others
PAWR	0.00336269	5.70046	Compound up vs All Others
MPHOSPH8	0.00336662	4.27292	Compound up vs All Others
HJURP	0.00337698	4.18473	Compound up vs All Others
GOLT1B	0.00338495	6.58454	Compound up vs All Others
DDX52	0.00341914	4.11707	Compound up vs All Others
RNF220	0.00342645	3.68295	Compound up vs All Others
HBP1	0.00343519	5.22299	Compound up vs All Others
RNMT	0.00343655	4.34516	Compound up vs All Others
COMMD7	0.0034381	5.14632	Compound up vs All Others
VAMP3	0.00343967	4.8199	Compound up vs All Others
DNAJC21	0.00344518	4.15172	Compound up vs All Others
PHC2	0.00349492	3.37486	Compound up vs All Others
PHB2	0.00349638	7.36292	Compound up vs All Others
CTDSPL	0.00350959	3.70147	Compound up vs All Others
ZFR	0.00352549	4.01484	Compound up vs All Others
KDM3B	0.00355076	4.01555	Compound up vs All Others
PPRC1	0.0035714	3.35776	Compound up vs All Others
HGSNAT	0.00357363	2.87575	Compound up vs All Others
KLHL28	0.0035921	3.84541	Compound up vs All Others
REPIN1	0.00361921	3.9381	Compound up vs All Others
BRD7	0.00365014	4.13348	Compound up vs All Others
PCNXL4	0.00365117	4.60747	Compound up vs All Others
CNOT2	0.00366153	3.87147	Compound up vs All Others
ANXA2	0.0036847	7.49714	Compound up vs All Others
RBMS1	0.00370209	3.53714	Compound up vs All Others
ATN1	0.00371325	2.8556	Compound up vs All Others
SLC25A6	0.00371441	3.50508	Compound up vs All Others
RMND5A	0.00371743	4.14138	Compound up vs All Others
RPS8	0.00374581	5.94733	Compound up vs All Others
SLC4A7	0.00374638	4.06217	Compound up vs All Others
THOP1	0.00374668	4.26479	Compound up vs All Others
SETD8	0.00377048	2.80297	Compound up vs All Others
RHBDF2	0.00378725	4.37732	Compound up vs All Others
RCC2	0.00379789	5.09946	Compound up vs All Others
FPGT	0.00380707	3.97575	Compound up vs All Others
CCNT2	0.00380945	4.04452	Compound up vs All Others
NSF	0.00381022	3.0604	Compound up vs All Others
AAGAB	0.00381619	5.96446	Compound up vs All Others
TAB3	0.00382793	3.14555	Compound up vs All Others
KIAA1033	0.00382973	3.08064	Compound up vs All Others
TRIM2	0.00383845	3.52784	Compound up vs All Others
SLC41A1	0.00384788	4.84658	Compound up vs All Others
PCNP	0.00384837	5.2213	Compound up vs All Others
ZFYVE20	0.00385403	3.43935	Compound up vs All Others
DUSP22	0.00387952	4.49259	Compound up vs All Others
C2orf49	0.003893	4.21934	Compound up vs All Others
GIGYF2	0.00390033	2.39583	Compound up vs All Others
C17orf49	0.00390136	7.21384	Compound up vs All Others
XIAP	0.00390824	3.09429	Compound up vs All Others
MLEC	0.00391323	2.11928	Compound up vs All Others
GMPS	0.00391623	5.94392	Compound up vs All Others
BIRC6	0.00392894	2.75653	Compound up vs All Others
EIF2AK4	0.00393365	3.53644	Compound up vs All Others
RBBP4	0.0039866	2.7993	Compound up vs All Others
NEMF	0.00401378	3.37796	Compound up vs All Others
COG5	0.00401598	4.09303	Compound up vs All Others
RPS5	0.00401974	5.59753	Compound up vs All Others
SLC39A14	0.00402516	3.68463	Compound up vs All Others
NFE2L3	0.00403287	4.92033	Compound up vs All Others
RAPH1	0.00403492	3.04428	Compound up vs All Others
PABPC4	0.00403563	4.09461	Compound up vs All Others
MAF	0.00406276	2.99305	Compound up vs All Others
NUP54	0.00408592	6.00731	Compound up vs All Others

ZNF768	0.00410144	5.04602	Compound up vs All Others
SPRY4	0.00413828	3.87502	Compound up vs All Others
BMS1P2	0.00414522	2.24457	Compound up vs All Others
BMS1P6	0.00414522	2.24457	Compound up vs All Others
MOB1A	0.00415661	7.00138	Compound up vs All Others
PRKAA1	0.00418185	3.86915	Compound up vs All Others
PCNA	0.00418271	7.52213	Compound up vs All Others
DNAJC8	0.00420514	6.29167	Compound up vs All Others
EIF4E	0.00422144	3.57011	Compound up vs All Others
MRFAP1	0.00422777	3.33568	Compound up vs All Others
FAM91A1	0.00423136	3.9179	Compound up vs All Others
WDR4	0.0042549	4.28511	Compound up vs All Others
FMNL2	0.00426853	4.08217	Compound up vs All Others
ARPC4	0.00428498	5.6438	Compound up vs All Others
UTP20	0.0042893	2.88581	Compound up vs All Others
PTAR1	0.00430156	3.36158	Compound up vs All Others
ACSL3	0.0043034	4.20001	Compound up vs All Others
CTBS	0.00432913	4.09172	Compound up vs All Others
VHL	0.00433639	2.6973	Compound up vs All Others
TRMT13	0.00437305	6.09571	Compound up vs All Others
GTF2I	0.00439581	4.36473	Compound up vs All Others
MARCKS	0.00443371	3.37468	Compound up vs All Others
LMAN1	0.00447818	3.61203	Compound up vs All Others
RCN1	0.0045047	4.14609	Compound up vs All Others
HBS1L	0.00452644	3.66752	Compound up vs All Others
LIN7C	0.00453444	4.62701	Compound up vs All Others
OLA1	0.00456168	4.20316	Compound up vs All Others
TMEM43	0.00458548	3.24708	Compound up vs All Others
ARPC5	0.00459173	5.35164	Compound up vs All Others
PKM	0.00462298	2.97861	Compound up vs All Others
RPS9	0.0046461	3.2891	Compound up vs All Others
PITPNA	0.00467884	3.52376	Compound up vs All Others
ITSN1	0.00469742	3.05534	Compound up vs All Others
HIST1H2BC	0.00470056	9.44829	Compound up vs All Others
TUBA1A	0.00471395	5.3769	Compound up vs All Others
EPN1	0.00472194	3.72198	Compound up vs All Others
PODXL2	0.00473597	4.40264	Compound up vs All Others
CHAMP1	0.00475613	2.69957	Compound up vs All Others
TYW1	0.00477212	4.74225	Compound up vs All Others
TUBGCP6	0.00482089	2.81962	Compound up vs All Others
CRK	0.00482225	4.45676	Compound up vs All Others
CLDN12	0.0048363	5.32353	Compound up vs All Others
NUP153	0.00486251	3.81751	Compound up vs All Others
MED25	0.00486721	3.167	Compound up vs All Others
CIRH1A	0.00488659	5.87868	Compound up vs All Others
OCLN	0.00489077	4.03925	Compound up vs All Others
FGFR1OP2	0.00489358	5.73537	Compound up vs All Others
TBRG4	0.00489755	5.23503	Compound up vs All Others
TRRAP	0.00490566	2.39175	Compound up vs All Others
STK24	0.00490931	2.79127	Compound up vs All Others
DNAJC13	0.00492303	3.54819	Compound up vs All Others
TMED7	0.00494442	5.69521	Compound up vs All Others
FNTA	0.00496472	5.39438	Compound up vs All Others
CCND1	0.00497725	3.98973	Compound up vs All Others
HIST1H2BE	0.00500286	12.3566	Compound up vs All Others
SS18L1	0.00502063	4.69169	Compound up vs All Others
GPI	0.0050385	3.09541	Compound up vs All Others
ASF1A	0.00504269	5.98984	Compound up vs All Others
SSB	0.0050554	7.4799	Compound up vs All Others
CHRAC1	0.00514991	4.51065	Compound up vs All Others
PMEPA1	0.00516997	2.50047	Compound up vs All Others
PSD3	0.00519621	3.02948	Compound up vs All Others
TEX261	0.00520441	4.88407	Compound up vs All Others
PLEKHA8	0.00521108	3.04127	Compound up vs All Others
OSGIN2	0.00525146	3.84574	Compound up vs All Others

UAP1	0.00526153	5.0407	Compound up vs All Others
FBXO33	0.00526367	2.50334	Compound up vs All Others
RPS11	0.00527309	7.00793	Compound up vs All Others
RPL23	0.00528347	5.46114	Compound up vs All Others
POLA2	0.00534185	4.83948	Compound up vs All Others
ZNHIT1	0.00535662	7.16823	Compound up vs All Others
ERGIC2	0.00539034	4.82362	Compound up vs All Others
TMPO	0.00540307	2.7925	Compound up vs All Others
G6PC3	0.00541935	3.81426	Compound up vs All Others
ATP5J	0.00543401	5.00398	Compound up vs All Others
MYL12B	0.00543545	5.77472	Compound up vs All Others
VGLL4	0.00548744	2.38652	Compound up vs All Others
PTOV1	0.00551758	4.17193	Compound up vs All Others
PRPF38A	0.00553983	5.09439	Compound up vs All Others
NUP62	0.00557475	2.31583	Compound up vs All Others
TECR	0.00558491	6.0485	Compound up vs All Others
OTULIN	0.00559946	3.45446	Compound up vs All Others
FBLIM1	0.00560547	4.4214	Compound up vs All Others
NAE1	0.00560883	6.26459	Compound up vs All Others
NOLC1	0.00562745	4.87781	Compound up vs All Others
KRBA1	0.00563713	3.56954	Compound up vs All Others
CAPRIN1	0.0056556	3.267	Compound up vs All Others
RPAP2	0.00566418	5.19442	Compound up vs All Others
PPHLN1	0.00566952	4.30881	Compound up vs All Others
AGPAT5	0.00570892	3.30935	Compound up vs All Others
ADNP2	0.00573671	3.56624	Compound up vs All Others
UBE2D2	0.00575423	3.92882	Compound up vs All Others
EEF1B2	0.00576515	6.67614	Compound up vs All Others
FAM199X	0.00577457	2.87416	Compound up vs All Others
ELP2	0.00579613	3.87434	Compound up vs All Others
ZC3H15	0.00582837	4.50902	Compound up vs All Others
GLT8D1	0.0058449	3.8857	Compound up vs All Others
TMOD2	0.00584921	4.16982	Compound up vs All Others
PLEKHM3	0.00585153	2.96829	Compound up vs All Others
SH2B1	0.00589241	2.83985	Compound up vs All Others
SRSF9	0.00589697	4.31204	Compound up vs All Others
MFSD1	0.00592762	4.22454	Compound up vs All Others
ZNF326	0.00593012	3.64438	Compound up vs All Others
MORF4L2	0.005956	3.61834	Compound up vs All Others
ERI3	0.00596511	4.66112	Compound up vs All Others
TP53BP2	0.00597452	3.43994	Compound up vs All Others
TRMT2A	0.0059794	3.04296	Compound up vs All Others
CPNE3	0.00598604	4.01503	Compound up vs All Others
CEP250	0.00598674	2.63225	Compound up vs All Others
PGK1	0.00599284	4.04071	Compound up vs All Others
PSMC4	0.00599967	5.82172	Compound up vs All Others
CSNK1G3	0.00604384	4.17407	Compound up vs All Others
PC	0.00606759	2.80962	Compound up vs All Others
MOSPD2	0.00607661	4.42831	Compound up vs All Others
PYGB	0.00609642	3.38309	Compound up vs All Others
POLR3H	0.00610656	3.06113	Compound up vs All Others
SRPK2	0.0061374	3.16995	Compound up vs All Others
KRR1	0.00614072	5.1105	Compound up vs All Others
FOPNL	0.00614859	5.19137	Compound up vs All Others
BAZ1A	0.006155	3.73932	Compound up vs All Others
