# Supplementary material for: Profiling molecular regulators of recurrence in chemorefractory triple-negative breast cancers
Source: Breast Cancer Res. 2019 Aug 5;21:87. doi: 10.1186/s13058-019-1171-7 (PMC6683504; doi:10.1186/s13058-019-1171-7)
Supplement: Supplementary file 8 — Table S7. Effectors of p53 inactivation upstream regulators (PDF 199 kb) [file 13058_2019_1171_MOESM8_ESM.pdf]

Upstream Regulator	Predicted Activation State	Activation z-score	p-value of overlap
ESR1	Activated	3.235	0.000000132
CST5	Inhibited	-2.982	0.0000132
MYC	Activated	4.177	0.0000148
NTRK2	Activated	2.538	0.0000692
MYCN	Activated	2.584	0.000329
FOXM1	Activated	2.957	0.000594
E2F3	Activated	2.155	0.000904
NDRG1	Inhibited	-2.219	0.00122
CDKN2A	Inhibited	-2.239	0.00141
EIF4E	Activated	2.755	0.00175
ERBB2	Activated	2.307	0.00192
GAST	Activated	2.362	0.00267
XBP1	Activated	3.148	0.00391
TCF7L2	Activated	3.934	0.00476
E2F1	Activated	2.895	0.00534
AKT1	Activated	3.07	0.00668
HGF	Activated	2.957	0.00803
FN1	Activated	2.671	0.0105
NUPR1	Inhibited	-2.837	0.0108
RICTOR	Inhibited	-3.606	0.0113
SET	Inhibited	-2	0.0151
SP1	Activated	2.806	0.0244
MAPK3	Activated	2.236	0.0249
KLF3	Inhibited	-2.138	0.0322
BCR (complex)	Activated	2.027	0.0335
FLT1	Activated	2.236	0.0341
KDM5B	Inhibited	-2.02	0.0443
SYVN1	Activated	2.646	0.0458
ERG	Activated	2.236	0.0461
MTPN	Activated	2.2	0.0472
