# Supplementary material for: Profiling molecular regulators of recurrence in chemorefractory triple-negative breast cancers
Source: Breast Cancer Res. 2019 Aug 5;21:87. doi: 10.1186/s13058-019-1171-7 (PMC6683504; doi:10.1186/s13058-019-1171-7)
Supplement: Supplementary file 9 — Table S8. 18q21.1-21.2 expressed genes (PDF 186 kb) [file 13058_2019_1171_MOESM9_ESM.pdf]

Gene Symbol	Gene Name
MBD1	methyl-CpG binding domain protein 1
MBD2	methyl-CpG binding domain protein 2
SNORD58A	small nucleolar RNA, C/D box 58A
SNORD58B	small nucleolar RNA, C/D box 58B
SNORD58C	small nucleolar RNA, C/D box 58C
MYO5B	myosin VB
CTIF	cap binding complex dependent translation initiation factor
C18orf25	chromosome 18 open reading frame 25
POLI	DNA polymerase iota
PIAS2	protein inhibitor of activated STAT 2
SCARNA17	small Cajal body-specific RNA 17
SETBP1	SET binding protein 1
SMAD2	SMAD family member 2
SMAD4	SMAD family member 4
SMAD7	SMAD family member 7
TCF4	transcription factor 4
ACAA2	acetyl-CoA acyltransferase 2
RAB27B	RAB27B, member RAS oncogene family
MEX3C	mex-3 RNA binding family member C
