# Supplementary material for: Profiling molecular regulators of recurrence in chemorefractory triple-negative breast cancers
Source: Breast Cancer Res. 2019 Aug 5;21:87. doi: 10.1186/s13058-019-1171-7 (PMC6683504; doi:10.1186/s13058-019-1171-7)
Supplement: Supplementary file 10 — Table S9. Multivariate survival analysis statistics. p values are log-rank. (PDF 41 kb) [file 13058_2019_1171_MOESM10_ESM.pdf]

	Dependent Variable	Adjusted p-value RCB
TP53 Copy Number (Figure 4b)	DFS	0.05
TP53 Copy Number (Figure 4b)	OS	0.05
TP53 3-way (Figure 4c)	DFS	0.05
TP53 3-way (Figure 4c)	OS	0.02
Compound TP53 (Figure 5d)	DFS	0.03
Compound TP53 (Figure 5d)	OS	0.02
SMAD4 CNV (Figure 6b)	DFS	0.03

Adjusted p-value LN Status
0.02
0.02
0.009
0.005
0.003
0.002
0.02
